# Humoral responses to SARS-CoV-2 vaccine in vasculitis-related immune suppression

**DOI:** 10.1126/sciadv.adq3342

**Published:** 2025-02-12

**Authors:** Kimia Kamelian, Benjamin Sievers, Michael Chen-Xu, Sam Turner, Mark Tsz Kin Cheng, Mazharul Altaf, Steven A. Kemp, Adam Abdullahi, Kata Csiba, Dami A. Collier, Petra Mlcochova, Bo Meng, Rachel B. Jones, The CITIID-NIHR BioResource COVID-19 Collaboration, Derek Smith, John Bradley, Kenneth G. C. Smith, Rainer Doffinger, Rona M. Smith, Ravindra K. Gupta

**Affiliations:** ^1^ School of Clinical Medicine, Department of Medicine, University of Cambridge, Cambridge, Cambridgeshire, UK.; ^2^ Cambridge Institute of Therapeutic Immunology and Infectious Disease (CITIID), Cambridge, Cambridgeshire, UK.; ^3^ Cambridge University Hospitals NHS Foundation Trust, Hills Road, Cambridge, Cambridgeshire, UK.; ^4^ Center for Pathogen Evolution, Department of Zoology, University of Cambridge, Cambridge CB2 3EJ, UK.; ^5^ Department of Renal Medicine, Addenbrooke’s Hospital, Cambridge, Cambridgeshire, UK.; ^6^ The Walter and Eliza Hall Institute of Medical Research (WEHI), Parkville, VIC 3052, Australia.; ^7^ University of Melbourne, Melbourne, VIC 3010, Australia.; ^8^ Department of Clinical Biochemistry and Immunology, Addenbrooke’s Hospital, Cambridge, UK.; ^9^ Africa Health Research Institute, Durban, South Africa.

## Abstract

Immune suppression poses a challenge to vaccine immunogenicity. We show that serum antibody neutralization against SARS-CoV-2 Omicron descendants was largely absent post-doses 1 and 2 in individuals with vasculitis treated with rituximab. Detectable and increasing neutralizing titers were observed post-doses 3 and 4, except for XBB. Rituximab in vasculitis exacerbates neutralization deficits over standard immunosuppressive therapy, although impairment resolves over time since dosing. We observed discordance between detectable IgG binding and neutralizing activity specifically in the context of rituximab use, with high proportions of individuals showing reasonable IgG titer but no neutralization. ADCC response was more frequently detectable compared to neutralization in the context of rituximab, indicating that a notable proportion of binding antibodies are non-neutralizing. Therefore, use of rituximab is associated with severe impairment in neutralization against Omicron descendants despite repeated vaccinations, with better preservation of non-neutralizing antibody activity.

## Teaser

SARS-CoV-2 vaccines induce suboptimal neutralizing antibody responses in individuals with vasculitis receiving rituximab.

## INTRODUCTION

COVID-19 claimed ~15.9 million lives globally in 2020 and 2021 (*1*), although nearly 20 million deaths were estimated to be prevented from vaccines (*2*). Severe acute respiratory syndrome coronavirus 2 (SARS-CoV-2) vaccine breakthrough infection in immunocompromised individuals is associated with persistent shedding and prolonged infections, resulting in highly mutated viral progeny that may select mutations and result in variants of concern (*3*–*5*). Therefore, protecting immunocompromised individuals from COVID-19 is critical at the level of public as well as individual health. Given that neutralizing antibodies are considered a surrogate for protection from SARS-CoV-2 infection (*6*–*8*) and clearance of infection (*9*), B cell–depleting agents represent a major challenge that remains under studied in patients with vasculitis in the Omicron phase of the COVID-19 pandemic.

Additional vaccinations following primary vaccination are recommended (*10*–*12*), and while they are associated with increased seroconversion as determined by immunoglobulin G (IgG)–binding antibodies (*13*, *14*), studies performed in the pre-Omicron era show lower seroconversion rates in immunocompromised individuals compared to the immunocompetent (*15*). Although most of immunocompromised individuals have received three or more doses of SARS-CoV-2 vaccinations, they bear a disproportionate disease burden, accounting for more than 20% of hospitalizations, admissions to intensive care units, and overall deaths associated with COVID-19 (*16*).

Very limited longitudinal vaccine immunogenicity studies with serum neutralization exist for immunocompromised individuals in the post-Omicron era, particularly those diagnosed with systemic vasculitis treated with immunosuppressing agents. Treatments for vasculitis rely on immunosuppressive therapies such as steroids and cytotoxic agents. Treatments of particular concern to vaccine immunogenicity are B cell–depleting antibodies such as rituximab. Rituximab is a humanized chimeric anti-CD20 monoclonal antibody that induces elimination of CD20 antigen–containing cells such as those located on cells of the B cell lineage (*17*), debilitating humoral immunity, and is used for the treatment of malignancies and autoimmune conditions (*18*).

**Fig. 1.**
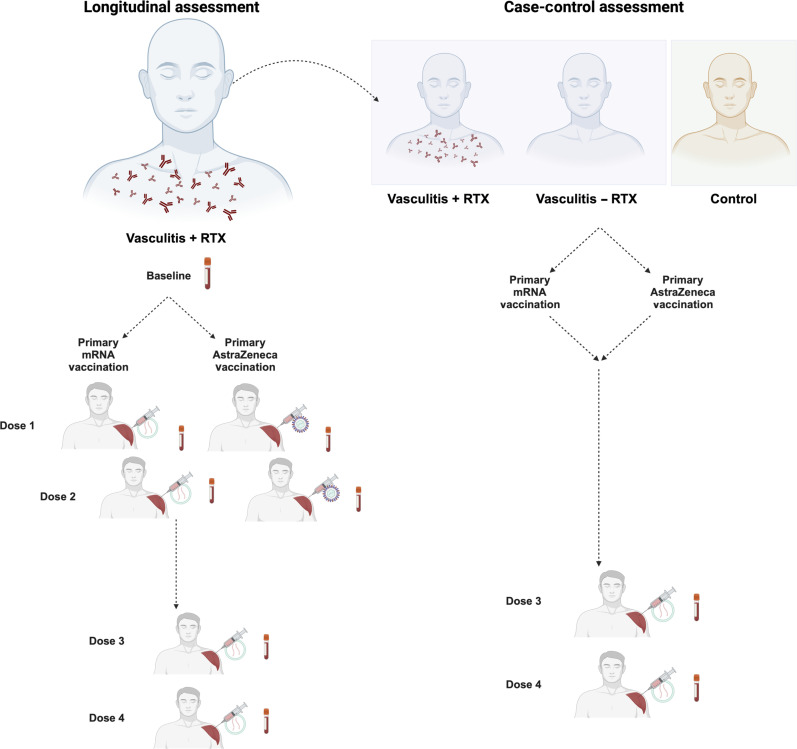
Schematic overview of study design: Longitudinal and case-control components. Individuals diagnosed with vasculitis with a history of rituximab (+RTX) (*n* = 197) within the past 5 years were identified. Individuals were stratified by primary vaccination group having received either two doses of BNT162b2 vaccine (Pfizer-BioNTech) (mRNA) (*n* = 73) or adenovirus-based AZD1222/ChAdOx1 nCoV-19 (AstraZeneca-Oxford) (AZN) (*n* = 114) vaccine. Longitudinal assessment: Individuals within each vaccination group were further identified if they had two additional mRNA boosters as part of routine clinical care and available samples from baseline and 1 month after doses 1 to 4 (AZN primary two dose course *n* = 23; and mRNA two dose primary course, *n* = 9). Neutralizing titers against WT, BA.1, BA.2, BA.4, and XBB were compared at baseline, post-dose 1, post-dose 2, post-dose 3, and post-dose 4 vaccination. Case-control assessment: Individuals originally diagnosed with vasculitis and a history of rituximab (+RTX) (*n* = 197) were included in this sub-study if they had available samples 1 month after doses 3 and 4 (*n* = 64). These individuals were age and sex matched with individuals diagnosed with vasculitis without a history of rituximab (*n* = 31) and with immunocompetent controls (*n* = 48). Neutralizing titers against WT, BA.1, BA.2, BA.4, and XBB were compared at post-dose 3 and post-dose 4 vaccination. Individuals were also stratified by primary vaccination type having received either two doses of mRNA or AZN vaccine, followed by two additional mRNA boosters as part of routine clinical care.

This study aimed to investigate longitudinal neutralizing antibody responses against SARS-CoV-2 in vaccinated individuals diagnosed with vasculitis and prescribed rituximab, representing a severely at-risk population. Initially, this study characterized longitudinal neutralizing titers, both before and following each of four successive vaccinations (Fig. 1). Subsequently, a case-control analysis was conducted to evaluate neutralizing and antibody-dependent cell-mediated cytotoxicity (ADCC) responses. Breadth of responses was measured against Omicron-descendent viruses, specifically distinguishing between those subjected to rituximab treatment and those without rituximab intervention, alongside immunocompetent individuals.

## RESULTS

### Characteristics of study participants

One hundred ninety-seven immunocompromised individuals diagnosed with vasculitis and treated with rituximab (+RTX) in the past 5 years were identified in the cohort (table S1). One hundred fourteen individuals (58%; males, 53%) with a median age of 59 years (25th to 75th percentile, 44 to 71) received adenovirus-based AZD1222/ChAdOx1 nCoV-19 (AstraZeneca-Oxford) (AZN) primary vaccines and 73 individuals (37%; males, 49%) with a median age of 63 years (46 to 71) received BNT162b2 vaccine (Pfizer-BioNTech) (mRNA) primary vaccines.

We sub-selected individuals with available samples pre-vaccination, post-dose 1, post-dose 2, post-dose 3, and post-dose 4 vaccination, comprising 23 and 9 individuals in the AZN and mRNA primary vaccination groups, respectively (Table 1). Comparative analysis of individuals between each primary vaccination group revealed similar median age [AZN 61 (50 to 71) and mRNA 63 (51 to 68)]. No significant differences were identified between comorbidities, renal diagnosis, estimated glomerular filtration rate (eGFR) stage, immunosuppressive treatments, and rituximab or cyclosporine course, although a nonsignificant difference in sex ratios [AZN males (61%) and mRNA males (33%)] was observed (Table 1). Median time between vaccine doses was 119 days (104 to 156) [AZN, 121 days (103 to 147); and RNA, 116 (107 to 163)], and samples were taken a median of 32 days (31 to 35) [AZN, 33 days (31 to 36); and RNA, 31 days (30 to 32)] after vaccination.

**Table 1.** Characteristics of individuals with primary AstraZeneca or mRNA COVID-19 vaccination in longitudinal cohort. AAV, ANCA-associated vasculitis; GPA, Granulomatosis with polyangiitis; MPA, Microscopic polyangiitis; FSGS, Focal segmental glomerulosclerosis; SLE, Systemic lupus erythematosus; AZA, Azathioprine; BEL, Belimumab; HCQ, Hydroxychloroquine; MMF, Mycophenolate mofetil; TAC, Tacrolimus.

Characteristic, no. (%), unless otherwise specified	AstraZeneca primary vaccine (*n* = 23)	mRNA primary vaccine (*n* = 9)	Total (*n* = 32)	*P* value
Baseline				
Age, years, median (Q1 to Q3)^*^	61 (50–71)	63 (51–68)	62 (50–71)	0.90^‡^
Sex^†^				
Male	14 (61)	3 (33)	17 (53)	0.24^§^
Female	9 (39)	6 (67)	15 (47)	
Comorbidities				
Hypertension	13 (57)	3 (33)	16 (50)	0.65^§^
Diabetes	3 (13)	2 (22)	5 (16)	
Chronic lung disease	8 (35)	2 (22)	10 (31)	
Cardiac disease	6 (26)	1 (11)	7 (22)	
Malignancy	1 (4)	1 (11)	2 (6)	
Cell count				
White cell count (10^4^), median (Q1 to Q3)				
Post-dose 3	7.8 (6.7–9.0)	8.9 (7.6–11)	8.0 (7.4–9.4)	0.14
Post-dose 4	7.8 (6.6–8.9)	8.0 (7.0–12)	7.9 (6.8–9.2)	0.28
Lymphocyte count (10^4^), median (Q1 to Q3)				
Post-dose 3	1.5 (1.1–1.7)	1.0 (0.6–1.3)	1.3 (0.9–1.6)	0.05
Post-dose 4	1.4 (1.2–1.8)	1.5 (0.9–1.6)	1.4 (1.2–1.7)	0.61
Renal diagnosis				
AAV	18 (78)	9 (100)	27 (84)	0.50
GPA	0 (0)	0 (0)	0 (0)	
MPA	0 (0	0 (0)	0 (0)	
FSGS	0 (0)	0 (0	0 (0)	
SLE	4 (17)	0 (0)	4 (13)	
Other	1 (4)	0 (0)	1 (3)	
eGFR stage				
1	9 (39)	3 (33)	12 (38)	0.41
2	6 (26)	5 (56)	11 (34)	
3	6 (26)	1 (11)	7 (22)	
4	2 (9)	0	2 (6)	
5	0	0	0	
Prednisolone				
+	10 (44)	5 (56)	15 (47)	0.62
−	13 (57)	4 (44)	17 (53)	
Immune sessions				
1	12 (52)	1 (11)	13 (41)	1.00
2	2 (9)	0 (0)	2 (6)	
3	1 (4)	0 (0)	1 (3)	
Immunosuppressives				
ANTI-IL5	3 (13)	0 (0)	3 (9)	1.00
AZA	2 (9)	0 (0)	2 (6)	
BEL	3 (13)	0 (0)	3 (9)	
HCQ	3 (13)	0 (0)	3 (9)	
MMF	3 (13)	1 (13)	4 (13)	
TAC	1 (4)	0 (0)	1 (3)	
Rituximab dose				
1	2 (9)	0 (0)	2 (6)	0.26
2	4 (17)	0 (0)	4 (13)	
3	4 (17)	1 (11)	5 (16)	
4	1 (4)	2 (22)	3 (9)	
5	5 (22)	1 (11)	6 (19)	
6	2 (9)	1 (11)	3 (9)	
7	1 (4)	3 (33)	4 (13)	
8	4 (17)	1 (11)	5 (16)	
Cyclosporine dose				
1	1 (4)	1 (11)	2 (6)	0.71
2	0 (0)	0 (0)	0 (0)	
3	0 (0)	0 (0)	0 (0)	
4	0 (0)	0 (0)	0 (0)	
5	2 (9)	1 (11)	3 (9)	
6	0 (0)	0 (0)	0 (0)	
7	0 (0)	1 (11)	1 (3)	
8	0 (0)	0 (0)	0 (0)	
9	0 (0)	0 (0)	0 (0)	
10	0 (0)	1 (11)	1 (3)	
11	2 (9)	0 (0)	2 (6)	

*Interquartile range.

†Sex was reported by participants.

‡Wilcoxon rank sum test.

§Fisher’s exact test.

Next, we performed a cross-sectional matched case-control study to assess the impact of rituximab on neutralizing titers and identified individuals diagnosed with vasculitis with and without a history of rituximab (+RTX, *n* = 64; and −RTX, *n* = 31) with samples available post-dose 3 and post-dose 4 vaccination (Table 2). No significant difference other than renal diagnosis (*P* < 0.05), immunosuppressive treatments (*P* < 0.05), and white cell count post-dose 4 (*P* < 0.05) was found between +RTX and −RTX individuals. Median age [+RTX 66 (51 to 71) and −RTX 64 (58 to 74)] and sex ratio [+RTX males (58%) and −RTX males (52%)] across groups were comparable. Median time between vaccine doses was 118 days (100 to 157), and samples were taken a median of 32 days (29 to 35) after vaccination. A total of 48 immunocompetent individuals were included in the control group from the NBR118 Study. The majority of individuals were males (58%), and median age was 69 years (61 to 72). Median time between vaccine doses was 317 days (187 to 355), and samples were taken a median of 37 days (33 to 100) after vaccination.

**Table 2.** Characteristics of individuals with and without rituximab treatment. AAV, ANCA-associated vasculitis; Immune GN, Pauci-immune glomerulonephritis; LVV, Large vessel vasculitis; OSV, Other small vessel vasculitis; SLE, Systemic lupus erythematosus; AZA, Azathioprine; BEL, Belimumab; HCQ, Hydroxychloroquine; MMF, Mycophenolate mofetil; MTX, Methotrexate; MEPO, Mepolizumab; IFX, Infliximab; VEDO, Vedolizumab; TOC, Tocilizumab; TAC, Tacrolimus; CIC, Ciclosporin.

Characteristic, no. (%), unless otherwise specified	Vasculitis (+RTX)				Vasculitis (−RTX)				*P* value
	AZN primary vaccine (*n* = 32)	mRNA primary vaccine (*n* = 32)	Total (*n* = 64)	*P* value	AZN primary vaccine (*n* = 17)	mRNA primary vaccine (*n* = 14)	Total (*n* = 31)	*P* value	
Baseline									
Age, years, median (Q1 to Q3)^*^	64 (51–71)	66 (51–72)	66 (51–71)	0.74^‡^	64 (61–73)	66 (52–75)	64 (58–74)	0.89^‡^	0.47^‡^
Sex^†^									
Male	18 (56)	19 (59)	37 (58)	1.00^§^	7 (41)	9 (64)	16 (52)	0.30^§^	0.66^§^
Female	14 (44)	13 (41)	27 (42)		10 (59)	5 (36)	15 (48)		
Comorbidities									
Hypertension	17 (53)	16 (50)	33 (52)	0.88	8 (47)	6 (43)	14 (45)	0.90	0.53
Diabetes	6 (19)	4 (13)	10 (16)		1 (6)	1 (7)	2 (6)		
Chronic lung disease	13 (41)	11 (34)	24 (38)		7 (41)	4 (29)	11 (35)		
Cardiac disease	6 (29)	3 (9)	9 (14)		5 (29)	3 (21)	8 (26)		
Malignancy	1 (3)	2 (6)	3 (5)		0 (0)	1 (7)	1 (3)		
Cell count									
White cell count (10^4^), median (Q1 to Q3)									
Post-dose 3	7.6 (6.6–9.1)	7.9 (7.0–9.8)	7.7 (6.8–9.5)	0.41	7.6 (5.9–9.1)	7.0 (5.7–8.3)	7.0 (5.7–9.1)	0.76	0.12
Post-dose 4	7.8 (7.2–8.9)	7.6 (6.9–10.1)	7.8 (7.0–9.5)	0.79	6.7 (5.6–8.8)	6.2 (5.6–8.9)	6.4 (5.6–8.8)	0.78	*P* < 0.05
Lymphocyte count (10^4^), median (Q1 to Q3)									
Post-dose 3	1.5 (0.9–1.7)	1.4 (1.0–1.8)	1.4 (0.9–1.7)	0.81	0.8 (0.7–1.3)	1.2 (0.6–1.8)	0.9 (0.6–1.4)	0.69	0.05
Post-dose 4	1.4 (1.0–2.2)	1.3 (0.8–1.6)	1.3 (0.9–1.7)	0.24	1.1 (0.7–1.7)	1.1 (0.8–1.5)	1.1 (0.8–1.6)	0.86	0.19
Renal diagnosis									
AAV	26 (81)	27 (84)	53 (83)	0.92	9 (53)	6 (43)	15 (48)	0.09	*P* < 0.05
Behçet	0 (0)	1 (3)	1 (2)		0 (0)	1 (7)	1 (3)		
Immune GN	1 (3)	1 (3)	2 (3)		0 (0)	0 (0)	0 (0)		
LVV	0 (0)	0 (0)	0 (0)		5 (29)	0 (0)	5 (16)		
OSV	0 (0)	0 (0)	0 (0)		1 (6)	2 (14)	3 (10)		
SLE	1 (3)	2 (6)	3 (5)		0 (0)	1 (7)	1 (3)		
Other	4 (13)	1 (3)	5 (8)		2 (12)	4 (29)	6 (19)		
eGFR stage									
1	8 (25)	10 (31)	18 (28)	0.31	5 (29)	3 (21)	8 (26)	0.64	0.16
2	8 (25)	12 (38)	20 (31)		6 (35)	7 (50)	13 (42)		
3	11 (34)	7 (22)	18 (28)		2 (12)	2 (14)	4 (13)		
4	3 (9)	0 (0)	3 (5)		4 (24)	1 (7)	5 (16)		
5	2 (6)	3 (9)	5 (8)		0 (0)	1 (7)	1 (3)		
Prednisolone									
+	19 (59)	14 (44)	33 (52)	0.32	8 (47)	10 (71)	18 (58)	0.27	0.66
−	13 (41)	18 (56)	31 (48)		9 (53)	4 (29)	13 (42)		
Immune sessions									
1	9 (28)	6 (19)	15 (23)	1.00	13 (76)	10 (71)	23 (74)	1.00	1.00
2	1 (3)	1 (3)	2 (3)		2 (12)	2 (14)	4 (13)		
3	0 (0)	0 (0)	0 (0)		1 (6)	0 (0)	1 (3)		
Immunosuppressives									
ANTI-IL5	0 (0)	0 (0)	0 (0)	0.61	1 (6)	0 (0)	1 (3)	0.45	*P* < 0.05
AZA	3 (9)	0 (0)	3 (5)		1 (6)	4 (29)	5 (16)		
BEL	2 (6)	2 (6)	4 (6)		0 (0)	0 (0)	0 (0)		
HCQ	3 (9)	3 (9)	6 (9)		1 (6)	1 (7)	2 (6)		
MMF	2 (6)	2 (6)	4 (6)		3 (18)	3 (21)	6 (19)		
MTX	0 (0)	0 (0)	0 (0)		4 (24)	1 (7)	5 (16)		
MEPO	0 (0)	0 (0)	0 (0)		1 (6)	0 (0)	1 (3)		
IFX	0 (0)	0 (0)	0 (0)		2 (12)	1 (7)	3 (10)		
VEDO	0 (0)	0 (0)	0 (0)		1 (6)	0 (0)	1 (3)		
TOC	0 (0)	0 (0)	0 (0)		2 (12)	0 (0)	2 (6)		
TAC	0 (0)	0 (0)	0 (0)		0 (0)	1 (7)	1 (3)		
CIC	0 (0)	0 (0)	0 (0)		0 (0)	1 (7)	1 (3)		
Rituximab dose									
1	1 (3)	1 (3)	2 (3)	0.73	–	–	–	–	–
2	2 (6)	2 (6)	4 (6)		–	–	–		
3	3 (9)	4 (13)	7 (11)		–	–	–		
4	6 (19)	6 (19)	12 (19)		–	–	–		
5	10 (31)	5 (16)	15 (23)		–	–	–		
6	3 (9)	3 (9)	6 (9)		–	–	–		
7	1 (3)	5 (16)	6 (9)		–	–	–		
8	6 (19)	6 (19)	12 (19)		–	–	–		
Cyclosporine dose									
1	3 (9)	1 (3)	4 (6)	0.16	0 (0)	0 (0)	0 (0)	1.00	0.13
2	3 (9)	0 (0)	3 (5)		0 (0)	0 (0)	0 (0)		
3	0 (0)	0 (0)	0 (0)		0 (0)	0 (0)	0 (0)		
4	0 (0)	2 (6)	2 (3)		1 (6)	0 (0)	1 (3)		
5	1 (3)	1 (3)	2 (3)		3 (18)	2 (14)	5 (16)		
6	0 (0)	1 (3)	1 (2)		0 (0)	0 (0)	0 (0)		
7	0 (0)	1 (3)	1 (2)		0 (0)	1 (7)	1 (3)		
8	0 (0)	0 (0)	0 (0)		0 (0)	0 (0)	0 (0)		
9	0 (0)	0 (0)	0 (0)		0 (0)	0 (0)	0 (0)		
10	0 (0)	1 (3)	1 (2)		0 (0)	0 (0)	0 (0)		
11	1 (3)	0 (0)	1 (2)		0 (0)	0 (0)	0 (0)		
12	0 (0)	0 (0)	0 (0)		0 (0)	0 (0)	0 (0)		
13	0 (0)	0 (0)	0 (0)		1 (6)	0 (0)	1 (3)		

*Interquartile range.

†Sex was reported by participants.

‡Wilcoxon rank sum test.

§Fisher’s exact test.

### Neutralizing titers are undetectable after primary vaccination in longitudinal vasculitis+RTX despite detectable IgG binding antibodies

**Fig. 2.**
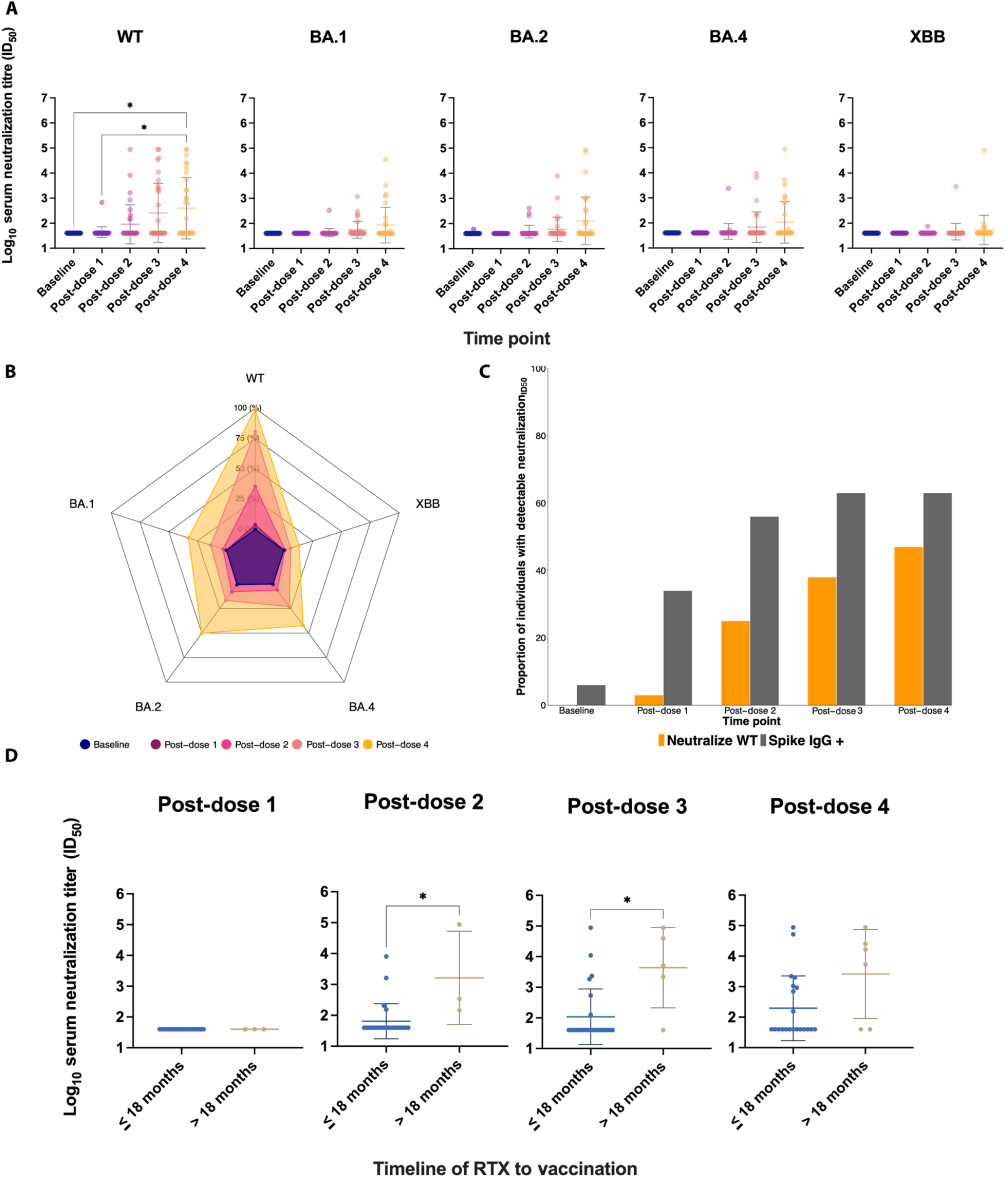
Impact of multiple SARS-CoV-2 vaccines on IgG binding and neutralizing antibody titers in longitudinal cohort. (**A**) Neutralizing titers significantly increased with additional vaccination doses (Freidman test: WT, *P* < 0.0001; BA.1, *P* < 0.0001; BA.2, *P* < 0.0001; and BA.4, *P* < 0.0001). This change was not as significant (*P* = 0.008) for XBB. (**B**) Radar plot demonstrating the neutralization breadth of variants, in comparison to a set maximum geometric mean titer (GMT) established from WT (100%), demonstrated increased GMT with additional vaccinations. (**C**) Proportion of individuals with neutralizing titers post-dose as compared to proportions with IgG binding antibodies. Post-dose 1 vaccination, while 34% of individuals had IgG binding antibodies, only 3.1% were capable of neutralizing WT representing an 11-fold discrepancy. (**D**) Individuals were administered one to three rituximab infusions by the end of the study period. Rituximab treatment within 18 months was associated with lower WT neutralization titers as compared to greater than 18 months before the vaccine dose (Kolmogorov-Smirnov test: post-dose 2, *P < 0.05; and post-dose 3, *P < 0.05). In (A), pairwise Dunn’s multiple comparison's test revealed significant differences between WT baseline and post-dose 4 titers (adjusted *P = 0.02), as well as between post-dose 1 and post-dose 4 titers (adjusted *P = 0.03).

Neutralizing activity was assessed among vasculitis+RTX individuals with available baseline and post-dose 1, post-dose 2, post-dose 3, and post-dose 4 samples (*n* = 32) using wild-type (WT), BA.1, BA.2, BA.4, and XBB pseudotyped viruses. Responses to WT were almost uniformly undetectable following dose 1 and dose 2 vaccination (Fig. 2, A to C). Detectable and increasing neutralizing titers were observed post-dose 3 and post-dose 4 vaccination (Fig. 2, A and B). Neutralizing titers to WT, BA.1, BA.2, and BA.4 variants significantly increased with additional vaccination (Freidman test, *P* < 0.0001 for each variant) (Fig. 2A). Although neutralizing titers to XBB also increased with additional vaccination, the magnitude was relatively small but anticipated given that XBB is a highly antigenically distinct variant (*19*) compared to WT (*P* = 0.008). The geometric mean titer (GMT) against WT increased nearly ninefold from 43.7 ± 1.6 (GMT ± SD) at post-dose 1 vaccination to 394.2 ± 16.7 (GMT ± SD) at post-dose 4 vaccination (Table 3). A smaller increase was seen with BA.1 [increased from 40.0 ± 1.1 (GMT ± SD) at post-dose 1 vaccination to 85.5 ± 5.1 (GMT ± SD) at post-dose 4 vaccination], BA.2 [increased from 40.0 ± 1.0 (GMT ± SD) at post-dose 1 vaccination to 126.9 ± 8.8 (GMT ± SD) at post-dose 4 vaccination], BA.4 [increased from 40.0 ± 1.0 (GMT ± SD) at post-dose 1 vaccination to 106.2 ± 6.7 (GMT ± SD) at post-dose 4 vaccination], and XBB [increased from 40.0 ± 1.0 (GMT ± SD) at post-dose 1 vaccination to 53.9 ± 3.8 (GMT ± SD) at post-dose 4 vaccination] (Table 3) (Fig. 2, A and B). Pairwise post hoc comparisons using Dunn’s multiple comparison’s test indicated significant differences between WT baseline and post-dose 4 titers (adjusted *P* = 0.02) as well as post-dose 1 and post-dose 4 titers (adjusted *P* = 0.03).

**Table 3.** Longitudinal geometric mean titers.

	Baseline	Post-dose 1	Post-dose 2	Post-dose 3	Post-dose 4
Total (*n* = 32)					
**WT**	40.0 ± 1.0	43.7 ± 1.6	90.7 ± 6.0	256.3 ± 15.2	394.2 ± 16.7
**BA.1**	40.0 ± 1.0	40.0 ± 1.0	42.8 ± 1.5	54.6 ± 2.2	85.5 ± 5.1
**BA.2**	40.5 ± 1.1	40.0 ± 1.0	48.0 ± 1.8	58.6 ± 3.0	126.9 ± 8.8
**BA.4**	40.0 ± 1.0	40.0 ± 1.0	45.8 ± 2.1	67.8 ± 4.1	106.2 ± 6.7
**XBB**	40.0 ± 1.0	40.0 ± 1.0	40.8 ± 1.1	45.7 ± 2.1	53.9 ± 3.8

Post-dose 1 vaccination, neutralizing activity against pseudotyped viruses was undetectable (Freidman test: baseline, *P* > 0.05; and post-dose 1, *P* > 0.05) (fig. S1). Post-dose 2, post-dose 3, and post-dose 4 vaccination showed significant differences in GMT (Freidman test: post-dose 2, *P* < 0.0001; post-dose 3, *P* < 0.0001; and post-dose 4, *P* < 0.0001). Pairwise post hoc comparisons using Dunn’s multiple comparison’s test indicated significant differences between WT and XBB 50% maximal inhibitory dilution (ID_50_) values post-dose 3 (adjusted *P* = 0.03) and post-dose 4 vaccination (adjusted *P* = 0.004), as well as for WT and BA.1 ID_50_ values post-dose 4 vaccination (adjusted *P* = 0.03), suggesting that, while boosters may increase neutralizing titers against WT, they have lower impact against newer variants and may not adequately protect in the setting of compromised immunity and rituximab treatment (fig. S1).

IgG binding antibodies were not reliable markers of neutralizing response in vasculitis+RTX individuals (Fig. 2C). This was evident postvaccinations when vaccine-induced IgG binding antibodies were detectable. For example, post-dose 1 vaccination, 34% of individuals had IgG binding antibodies, yet only 3.1% were capable of neutralizing WT representing an 11-fold difference. Still, there was an increase in the proportion of individuals with IgG binding antibodies and those with detectable neutralizing response with each additional vaccination. Post-dose 2 vaccination, 56% of individuals had IgG binding antibodies, and 25% had detectable WT neutralizing antibodies representing 2.3-fold difference. Post-dose 3 vaccination, 63% of individuals had IgG binding antibodies, and 38% had detectable WT neutralizing antibodies representing 1.7-fold difference. Post-dose 4 vaccination, 63% of individuals had IgG binding antibodies, and 47% had detectable WT neutralizing antibodies representing 1.3-fold difference. This discordant neutralizing response was more pronounced in the mRNA primary vaccination group (fig. S2A) regardless of natural infection (fig. S2B). There appears to be an observed lag in neutralizing responses in vasculitis+RTX relative to IgG binding responses that may be mitigated with additional vaccinations (Fig. 2C).

We previously reported differences between primary mRNA and adenovirus vaccine responses in the elderly, with the latter being associated with enrichment of atypical B cells after a third mRNA dose (*20*). mRNA vaccines have also been shown to generate higher neutralizing titer peaks in the short term as compared to adenovirus vaccines (*21*). We compared neutralizing responses based on the primary vaccination group in the longitudinal cohort comprising vasculitis+RTX individuals and while the *P* value was significant (Mann-Whitney test, *P* = 0.01) against WT post-dose 3 vaccination, at no other time point was pairwise comparison between vaccination groups statistically significant (fig. S3).

### Rituximab within 18 months of vaccination significantly impairs immunogenicity

To assess the potential impact of rituximab on vaccine immunogenicity, we examined longitudinal sera from individuals who received SARS-CoV-2 vaccinations after rituximab treatment. We specifically compared the relationship between neutralizing titers and the time elapsed between rituximab administration and serum collection. Individuals may have contributed data to more than one time point. We stratified participants by rituximab treatment in the last 18 months of vaccination, which revealed a significant difference in distribution of serum neutralizing titers for individuals with rituximab treatment within 18 months of the second and third vaccinations (Kolmogorov-Smirnov test: post-dose 2, *P* < 0.05; and post-dose 3, *P* < 0.05) (Fig. 2D).

### Immunocompromised status and rituximab treatment affect neutralization in matched case-control analysis

**Fig. 3.**
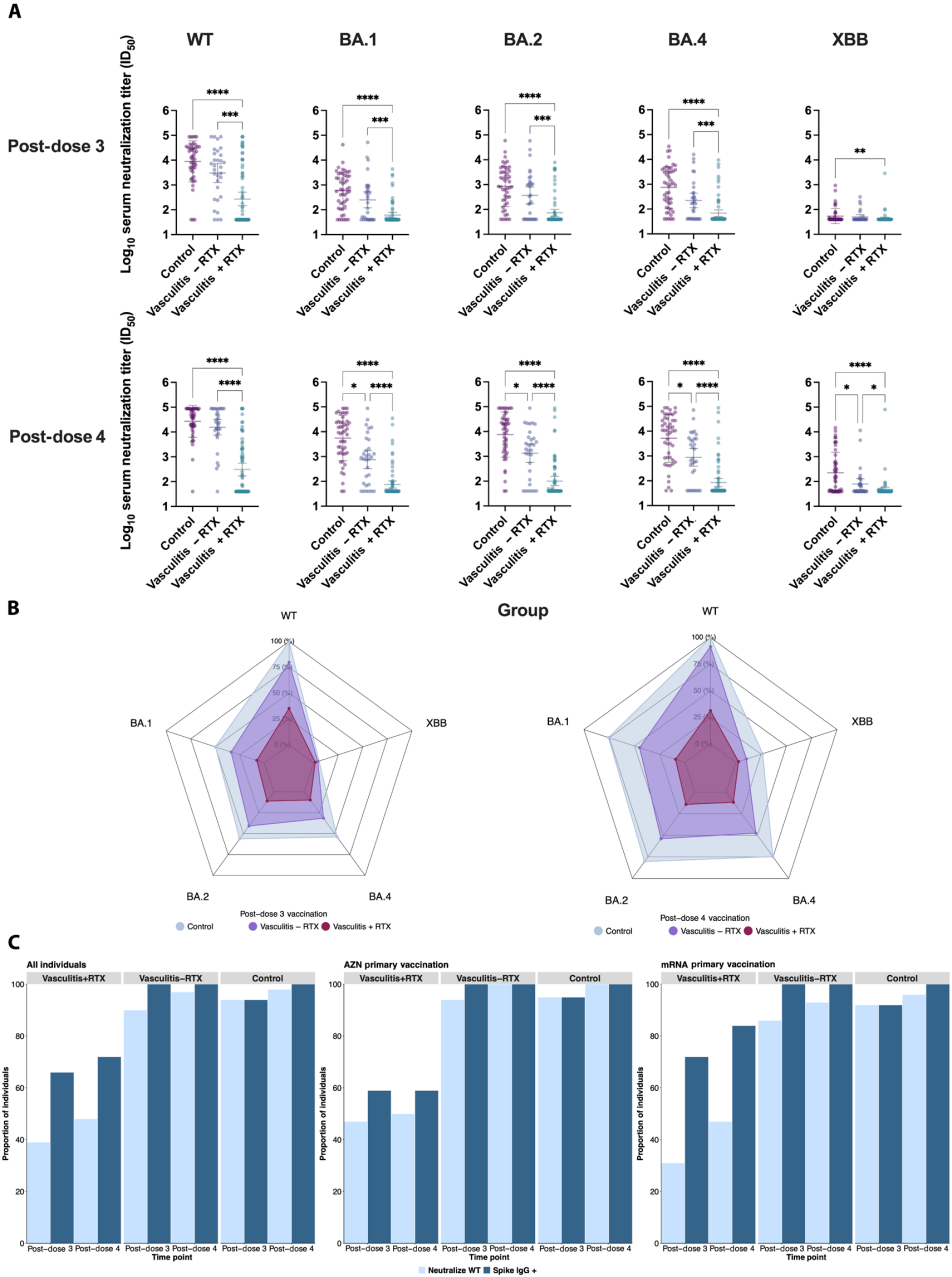
Impact of ±rituximab treatment on binding and neutralizing titers in case-control cohort. (**A**) Rituximab treatment (+RTX) severely affected neutralizing titers against pseudotyped viruses. Vasculitis+RTX had significantly lower titers than immunocompetent individuals post-dose 3 (Dunn’s test: ****P < 0.0001 for WT, BA.1, BA.2, and BA.4 and **P = 0.002 for XBB) and post-dose 4 (Dunn’s test: ****P < 0.0001 for WT, BA.1, BA.2, and BA.4 and *P = 0.02 for XBB) vaccination. Immunocompromised status became most significant post-dose 4 vaccination for neutralizing titers against non-WT SARS-CoV-2 variants (Dunn’s test: vasculitis−RTX versus control, 0.01 < *P* < 0.05). Rituximab treatment significantly affected neutralizing titers post-dose 3 (Dunn’s test: ***P < 0.001 for WT, BA.1, BA.2, and BA.4 and *P* = 0.30 for XBB) and post-dose 4 (Dunn’s test: ****P < 0.0001 for WT, BA.1, BA.2, and BA.4 and *P = 0.02 for XBB) vaccination relative to vasculitis−RTX. (**B**) Radar plots depict neutralization breadth against variants in individuals with vasculitis (±RTX), compared to a predefined maximum GMT established from the WT (100%). (**C**) Proportion of individuals with neutralizing titers post-doses 3 and 4 as compared to proportions with IgG binding antibodies, stratified by primary vaccination platform, shows discordance between the proportion of vasculitis+RTX individuals with detectable IgG binding and neutralizing antibodies (post-dose 3, 1.7-fold difference; and post-dose 4, 1.5-fold difference).

Neutralizing titers were compared between immunocompromised individuals diagnosed with vasculitis (*n* = 95) with (+RTX, *n* = 64) and without (−RTX, *n* = 31) rituximab treatment and immunocompetent individuals (*n* = 48). Rituximab treatment significantly affected neutralizing titers post-dose 3 (Kruskal-Wallis test: WT, *P* < 0.0001; BA.1, *P* < 0.0001; BA.2, *P* < 0.0001; BA.4, *P* < 0.0001; and XBB, *P* < 0.0025) and post-dose 4 (Kruskal-Wallis test, *P* < 0.0001 for each variant) vaccination (Fig. 3, A and B). Vasculitis+RTX individuals consistently had lower neutralizing titers relative to vasculitis−RTX individuals post-dose 3 (Dunn’s test: *P* < 0.001 for WT, BA.1, BA.2, and BA.4 and *P* = 0.30 for XBB) and post-dose 4 vaccination (Dunn’s test: *P* < 0.0001 for WT, BA.1, BA.2, and BA.4 and *P* = 0.02 for XBB) and immunocompetent individuals post-dose 3 (Dunn’s test: *P* < 0.0001 for WT, BA.1, BA.2, and BA.4 and *P* = 0.002 for XBB) and post-dose 4 vaccination (Dunn’s test: *P* < 0.0001 for WT, BA.1, BA.2, BA.4, and XBB) (Fig. 3A) regardless of the primary vaccination group (fig. S4). The neutralization breadth against variants in individuals with vasculitis (±RTX), compared to a predefined maximum GMT established from the immunocompetent response to WT (100%), revealed increasing GMT for vasculitis−RTX, but, to a lesser extent, for vasculitis+RTX between dose 3 and dose 4 vaccination across all pseudotyped viruses observed (Fig. 3B). However, while vasculitis−RTX individuals had three- and twofold lower titers post-dose 3 and post-dose 4 vaccination, respectively, against WT relative to immunocompetent individuals, vasculitis+RTX had 33- and 88-fold, respectively. A diagnosis of vasculitis (±RTX) became significant post-dose 4 vaccination (Fig. 3A), with vasculitis−RTX having significantly lower neutralizing titers against non-WT variants relative to immunocompetent individuals (Dunn’s test: 0.01 < *P* < 0.05). Neutralizing titers among vasculitis+RTX were reduced 10-fold post-dose 3 and 60- to 80-fold post-dose 4 against BA.1, BA.2, and BA.4 compared to immunocompetent individuals, suggesting that rituximab impairs WT vaccine-induced neutralization breadth.

### Discordance between IgG binding and neutralizing antibody titers

A discordance between the proportion of individuals with detectable IgG binding and neutralizing antibodies was observed in vasculitis+RTX individuals (*n* = 64) in the matched case-control analysis post-dose 3 and post-dose 4 vaccination (Fig. 3C). Overall, post-dose 3 vaccination, 66% of vasculitis+RTX individuals had IgG binding antibodies, and 39% had detectable WT neutralizing antibodies, indicating a 1.7-fold difference. This discordance decreased post-dose 4 vaccination where 72% of vasculitis+RTX individuals had IgG binding antibodies and 48% had detectable WT neutralizing antibodies, indicating a 1.5-fold difference. Vasculitis−RTX individuals (*n* = 31) did not demonstrate discrepancy between binding and neutralizing titers post-dose 3 or post-dose 4 vaccination. Post-dose 3 vaccination, 100% of individuals had IgG binding antibodies, and 90% had detectable WT neutralizing antibodies, indicating a 1.1-fold difference, while post-dose 4 vaccination, 100% of these individuals had IgG binding antibodies, and 97% had detectable WT neutralizing antibodies, indicating a negligible difference (1.0-fold difference).

Immunocompetent individuals (*n* = 48) consistently had elevated and concordant IgG binding and detectable neutralizing titers (Fig. 3C) post-dose 3 (IgG binding antibodies, 94%; and neutralizing antibodies, 94%) and post-dose 4 (IgG binding antibodies, 100%; and neutralizing antibodies, 98%) vaccinations relative to vasculitis (±RTX). All immunocompetent individuals primed with AZN (*n* = 22/22) and nearly all immunocompetent individuals primed with mRNA (*n* = 25/26) had accompanying detectable WT neutralizing titers post-dose 4 vaccinations.

Stratification by N-specific IgG binding antibodies (reflecting prior infection) indicated no significant difference in neutralizing response between primary vaccination type within the immunocompetent, vasculitis−RTX, and vasculitis+RTX groups (fig. S5, A to C). While neutralizing titers increased with additional vaccination, there was a lag in neutralizing response relative to IgG binding response for vasculitis+RTX individuals (fig. S5C). Nevertheless, prior detectable infection in vasculitis+RTX appeared to promote increased IgG binding response (fig. S5, A and B).

### ADCC response is better preserved compared to neutralization in vasculitis+RTX

**Fig. 4.**
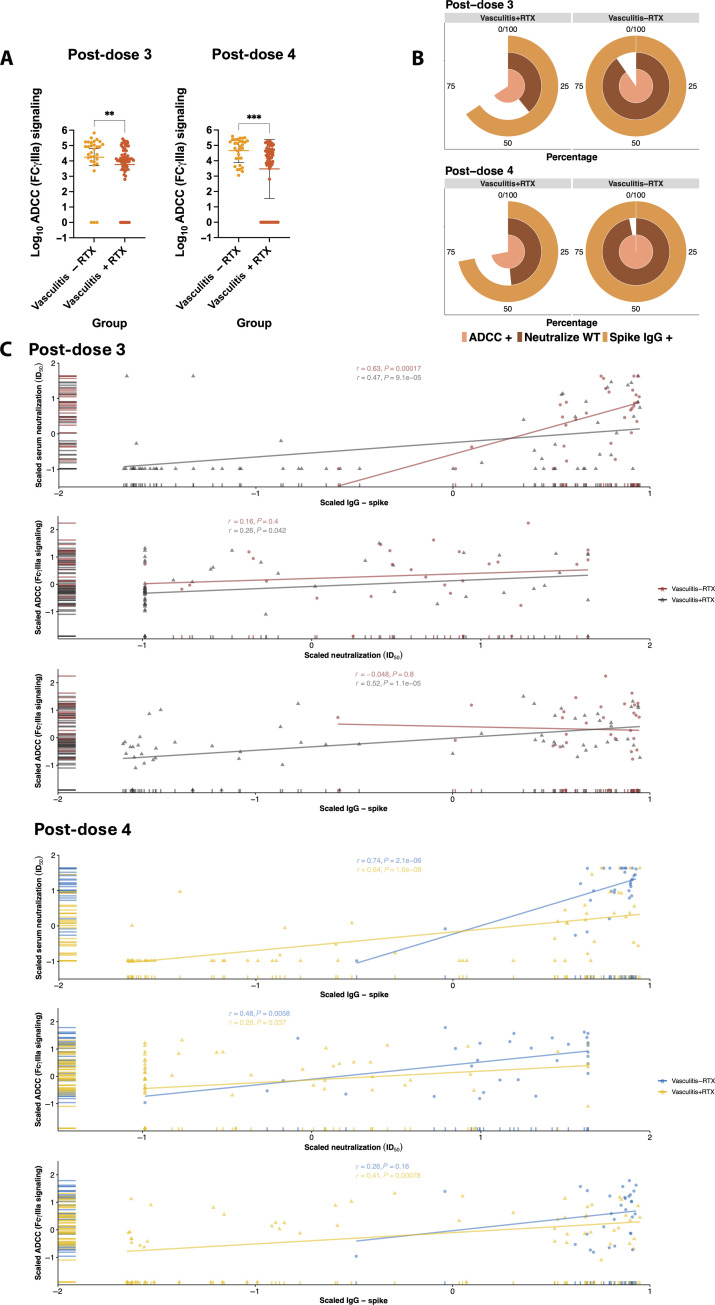
ADCC response apparent in vasculitis±RTX despite discordance between IgG binding and neutralizing antibodies. (**A**) ADCC response was significantly lower in vasculitis+RTX (GMT ± SD: post-dose 3, 5910.0 ± 29.5; and post-dose 4, 2954.0 ± 82.7) than vasculitis−RTX (GMT ± SD: post-dose 3, 17573 ± 33.7; and post-dose 4, 45995.0 ± 6.1) post-dose 3 (Mann-Whitney test, **P < 0.05) and post-dose 4 (Mann-Whitney test, ***P < 0.005) vaccination. (**B**) Most vasculitis+RTX have ADCC response, suggesting that the discrepancy identified between IgG binding antibodies and detectable neutralizing response may be accounted for by non-neutralizing binding antibodies. (**C**) There is a moderate positive correlation between IgG binding and neutralizing antibody titers post-dose 3 and post-dose 4 vaccination indicated through Pearson’s correlation (vasculitis−RTX, *r* = 0.47, *P* = 9.1 × 10^−5^; and vasculitis+RTX, *r* = 0.63, *P* = 0.00017). There is a weak to moderate correlation between ADCC and both neutralization and IgG binding responses regardless of rituximab treatment.

To determine whether a proportion of the non-neutralizing antibodies observed in vasculitis+RTX could have alternative antiviral activity, we assessed ADCC response to WT in the vasculitis group as vasculitis−RTX had comparable neutralizing and binding titers to immunocompetent individuals. ADCC response was significantly lower in vasculitis+RTX (GMT ± SD: post-dose 3, 5910.0 ± 29.5; and post-dose 4, 2954.0 ± 82.7) than vasculitis−RTX (GMT ± SD: post-dose 3, 17573 ± 33.7; and post-dose 4, 45995.0 ± 6.1) post-dose 3 (Mann-Whitney test, *P* < 0.05) and post-dose 4 (Mann-Whitney test, *P* < 0.005) vaccination (Fig. 4A). However, the majority of vasculitis−RTX contrary to vasculitis+RTX had detectable ADCC titers and detectable neutralizing titers post-dose 3 (ADCC, 90% versus 89%; and detectable neutralizing titers, 90% versus 39%) and post-dose 4 (ADCC, 100% versus 78%; and detectable neutralizing titers, 97% versus 48%) vaccination contrary to vasculitis+RTX (Fig. 4B). These data suggest that the discrepancy identified between IgG binding antibodies and detectable neutralizing response may be accounted for by binding yet non-neutralizing antibodies with ADCC activity. There was moderate positive correlation between IgG binding and neutralizing antibody titers against WT post-dose 3 [Pearson’s correlation: vasculitis−RTX: correlation coefficient (*r*) = 0.47, *P* = 9.1 × 10^−5^; and vasculitis+RTX: *r* = 0.63, *P* = 0.00017], and there was a slight increase in correlation post-dose 4 (Pearson’s correlation: vasculitis−RTX: *r* = 0.74, *P* = 2.1 × 10^−6^; and vasculitis+RTX: *r* = 0.64, *P* = 1.6 × 10^−8^) vaccination (Fig. 4C). There was weak to moderate correlation between ADCC and neutralization response and ADCC and IgG binding response for both vasculitis−RTX and vasculitis+RTX individuals.

A further investigation into the correlation between IgG binding and neutralizing antibodies was performed using an optimized generalized linear mixed model using gamma distribution and a log link function fit by maximum likelihood (fig. S6, A and B). Post-dose 3 vaccination, for each one-unit increase in IgG binding titer, the expected ID_50_ increases by 1.43 and 0.37% in vasculitis−RTX and vasculitis+RTX, respectively. Post-dose 4 vaccination, the expected ID_50_ increases remained stable (1.43% per unit change in binding IgG for vasculitis−RTX versus 0.42% for vasculitis+RTX). The SD was high (post-dose 3, 3.24; and post-dose 4, 2.32) at each time point, highlighting the significance for accounting of individual-specific effects. Rituximab treatment significantly affected expected ID_50_ titers compared to vasculitis−RTX (post-dose 3, *P* = 0.01; and post-dose 4, *P* = 0.02), and IgG binding titer was also significantly affected by rituximab treatment (post-dose 3, *P* < 0.001; and post-dose 4, *P* = 5.45e-05). The interactive effect of IgG binding titer and rituximab treatment was different for vasculitis+RTX relative to vasculitis−RTX (post-dose 3, *P* = 0.01; and post-dose 4, *P* = 0.005).

### Antibody landscapes in individuals with vasculitis and impact of rituximab

**Fig. 5.**
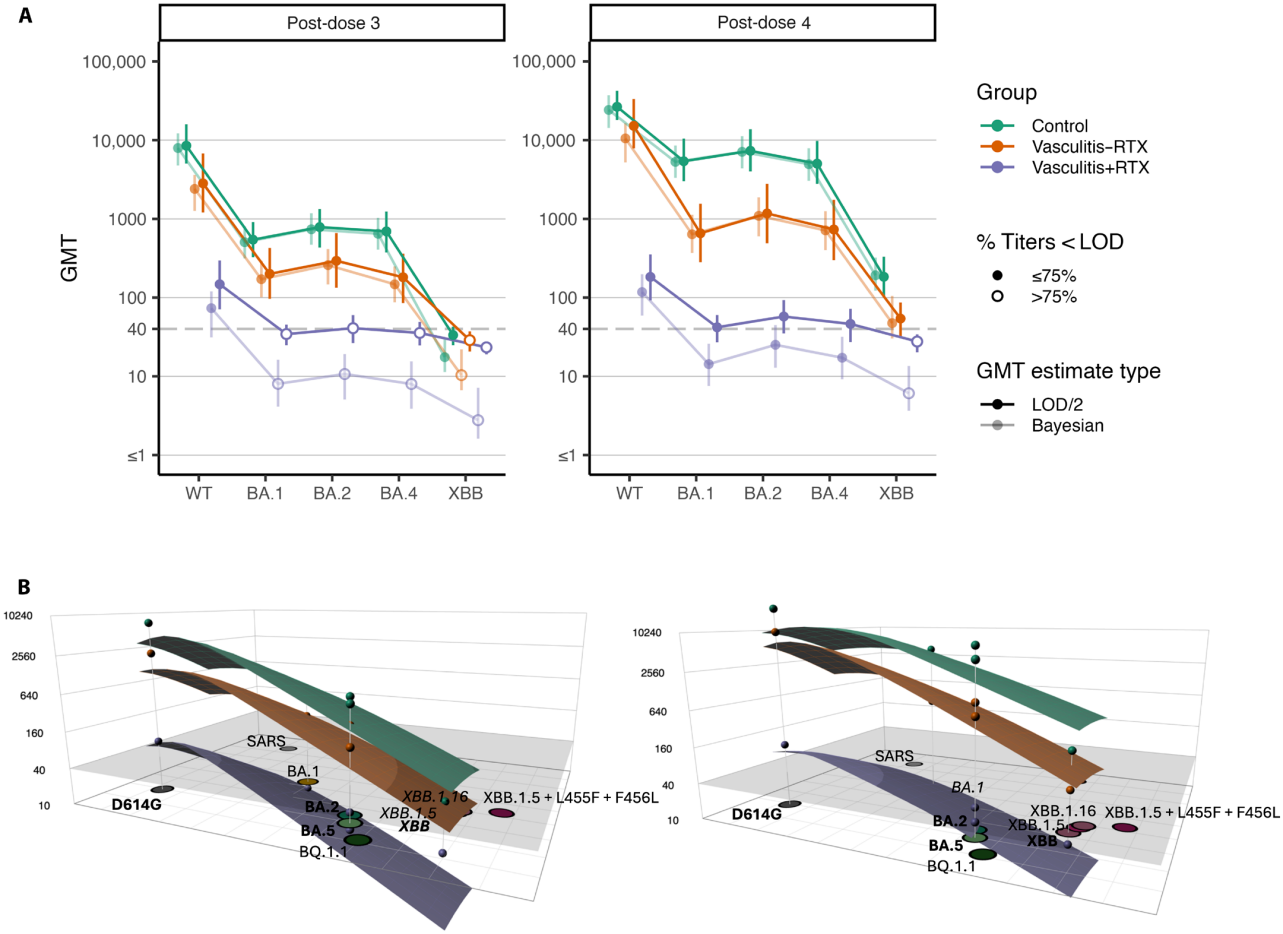
Estimated titers plots and antibody landscapes highlight extensive differences in responses to variants based on immune status and use of rituximab. (**A**) Titers plots for post-dose 3 and post-dose 4 sera from the control, vasculitis−RTX, and vasculitis+RTX groups. GMTs were calculated by two methods: (i) by treating titers below limit of detection (LOD) as 50% of the lower limit of detection and (ii) using a maximum likelihood estimation method. (**B**) Antibody landscapes for control, vasculitis−RTX, and vasculitis+RTX for post-dose 3 and post-dose 4 vaccination sera. The gray horizontal plane represents the limit of detection, and circular points represent the maximum likelihood GMT values for each group against each variant. The landscapes were fitted to an antigenic map in which the circles represent different variants, which was produced using mouse antiserum titrations. Variants are written in bold if human serum titrations are available against the variant (and it is, therefore, used in fitting the landscape), and in italics if the circle representing the variant is hidden by the landscape.

Last, we generated antibody landscapes to visualize the serum neutralization profiles for control, vasculitis−RTX, and vasculitis+RTX participants from the case-control study. Antibody landscapes represent the neutralization profile of a serum group as a surface in the third dimension above an antigenic map, which shows the antigenic relationship between variants in two-dimensional space. We first plotted GMTs for risk groups against each variant with post-dose 3 and post-dose 4 sera (Fig. 5A). Antibody landscapes (Fig. 5B) are shown for the post-dose 3 and post-dose 4 sera from the control, vasculitis−RTX, and vasculitis+RTX groups. The landscapes show a reduced neutralization profile across variants for vasculitis−RTX compared to immunocompetent controls and a substantial reduced neutralization profile for vasculitis+RTX compared to vasculitis−RTX. At post-dose 4 vaccination, this difference widens rather than contracts, indicating that a defective B cell response from rituximab treatment cannot simply be overcome by further vaccination with WT vaccines. While immunological shortcomings in serum neutralization among vasculitis−RTX individuals can be improved with additional vaccinations, neutralizing response remained near the limit of detection for vasculitis+RTX.

## DISCUSSION

Immunocompromised individuals have diminished immunogenicity in response to SARS-CoV-2 vaccinations, in part, due to immunomodulating agents (*13*, *22*, *23*) such as rituximab (*24*, *25*). In this study, we find seroconversion is not indicative of neutralizing ability in immunocompromised individuals and should not be used equally as means of clinical significance as there were seroconverted individuals that were unable to neutralize WT post-dose 3 and post-dose 4 vaccination.

A third vaccination was required to detect neutralizing activity in individuals with vasculitis after primary vaccination for all pseudotyped viruses (WT, BA.1, BA.2, BA.4, and XBB). This finding was most evident for WT and least apparent for XBB where responses to the latter were uniformly poor. These results echo previous findings using similar approaches (*26*, *27*), suggesting that boosters, including homologous WT vaccines, can promote B cell differentiation and affinity maturation in individuals with previous vaccinations (*28*–*30*). This phenomenon occurs because, although long-lived plasma B cells produced during initial vaccination or infection secrete highly specific antibodies that may not have specificity or affinity for antigens from previously unencountered variants, memory B cells have broader range of antigen specificity and affinity and are also activated upon antigen rechallenge. Activated memory B cells can differentiate into long-lived plasma B cells with additional specificity and affinity against new antigens (*31*, *32*). As observed within our study and others (*33*), prior vaccination may not provide protection against variants with mutations in key epitope regions of vaccine-induced and therapeutic monoclonal antibodies. Pairwise post hoc comparisons indicated significant differences between WT and XBB ID_50_ values post-dose 3 and post-dose 4 vaccinations, suggesting that new variants may hinder the longitudinal immunogenicity of boosters. Variants such as XBB contain several mutations including R346T in the receptor binding domain of the spike protein that is associated with evasion of vaccine-induced neutralizing antibodies (*34*) and therapeutic monoclonal antibodies (*35*, *36*). Hence, individuals treated with rituximab are particularly vulnerable to antigenically distinct variants due to impaired germinal center reactivity and somatic hypermutation following vaccination with WT vaccines.

While there was an observed delay in neutralizing responses relative to increasing IgG binding responses for vasculitis+RTX individuals with successive vaccine doses, this discordance appeared to be only partially mitigated with additional vaccinations. The relatively severe impairment in neutralizing responses may obfuscate inferred vaccine-induced protection as binding antibodies obtained through serological assays (*37*) are frequently used as surrogate markers to assess viral neutralization.

Reduced humoral immunity is particularly concerning to vaccine immunogenicity in individuals treated with B cell–depleting agents. This is of relevance to public health as intra-host evolution within immunocompromised individuals that have difficulty clearing SARS-CoV-2 infection is thought to be a source for immune-escape variants (*4*, *38*). While CD20 antigen–containing B cells in the peripheral blood are depleted after rituximab treatment, the extent to which rituximab depletes CD20 antigen–containing B cells in solid tissues, such as germinal centers, can be dose dependent and specific to location of B cell microenvironment (*39*–*41*). While some have observed that rituximab-treated individuals recover from COVID-19 without severe disease (*42*), others have found a strong link between rituximab treatment and severity of COVID-19 disease (*25*, *43*) as well as suboptimal neutralizing titers postvaccinations (*44*, *45*). Data form the basis of use of neutralization titer as a proxy for protection. Shorter duration between rituximab treatment and COVID-19 vaccination appeared to impede neutralizing titers postvaccinations within our study. We found an increasing number of vasculitis+RTX individuals able to mount detectable neutralizing titers with additional vaccinations, suggesting that, while rituximab treatment may delay the emergence of detectable neutralizing titers, boosting can stimulate B cell responses.

Expectedly, the vasculitis+RTX individuals consistently displayed lower neutralizing titers relative to vasculitis−RTX and immunocompetent individuals’ post-dose 3 and post-dose 4 vaccination. Responses between vasculitis−RTX and immunocompetent individuals were comparable post-dose 3 vaccination but became significantly different post-dose 4 vaccination against non-WT variants, possibly due to the additive impact of natural non-WT infection in immunocompetent individuals; although proportionately, responses did not differ between those with and without prior infection. Our responses are similar to others looking at the impact of immunomodulating agents such as Cheung *et al.* (*46*) reporting that individuals with impaired immunity treated with anti–tumor necrosis factor have a deficit in postvaccination response relative to other immunocompromised individuals. Boosting with the WT strain in the presence of new variants provides short-lived protection in immunocompetent individuals (*47*). Immunocompromised individuals face additional challenges as they are unable to develop initial humoral responses following primary vaccination, including intact well-defined germinal centers (*44*). Boosting with lineage-specific vaccines can lead to diversification of previously selected B cells in immunocompetent individuals (*48*, *49*) and could promote further expansion of an albeit limited but established B cell population (*44*) in immunocompromised individuals. Further studies are urgently needed to address this.

We identified a discordance between neutralizing and IgG binding antibody titers, mainly attributed to vasculitis+RTX individuals primed with mRNA vaccines, signifying the potential impact of rituximab on vaccine-induced response and immunological memory. We speculate that the longer duration of antigenic stimulation associated with adenovirus vaccines may favor generation of more mature neutralizing S-specific antibodies. In addition, we assessed ADCC response to WT in vasculitis±RTX to investigate whether the discrepancy between IgG binding and neutralizing titers was possibly due to non-neutralizing antibodies. While there were significant differences in median response, most of vasculitis+RTX had an ADCC response. Serological assays, therefore, may not reliably reflect neutralizing titers particularly in the presence of rituximab treatment.

Our study is limited by a modest longitudinal sample set. Still, to our knowledge, this is the largest study to date investigating the impact of longitudinal vaccinations in individuals diagnosed with vasculitis using neutralization against a range of Omicron-descendant variants (*50*, *51*). We did not control for additional treatments such as cyclosporine and prednisolone. While this limitation may affect our ability to draw definitive conclusions about the impact of treatment combinations on detectable neutralizing titers, individuals treated with rituximab are often treated with a diverse set of regions, and we believe that the study population is reflective of the real-world treatment landscape. In addition, the temporal relationship between time since RTX and the impairment in neutralization indicates that the RTX is primarily responsible for neutralization defect. The sample set is constrained by lack of available peripheral blood mononuclear cells, and, as such, we are limited in our capacity to examine T cell responses or B cell repertoires to assess clonal expansion of receptor binding domain–specific B cells. Breakthrough infection aside from history of N-positive antibody titers at time of sample collection was not included in this study as individuals did not undergo regular COVID-19 testing to screen for asymptomatic infection.

Our results have significant implications for individuals treated with rituximab in the post-Omicron era, highlighting the value of additive boosters in affirming increasing protection in clinically vulnerable populations. The use of rituximab is associated with severe impairment of neutralization against Omicron-descendant variants, highlighting the urgent need for additional adjunctive strategies to enhance vaccine-induced immunity as well as preferential access for such patients to updated vaccines using spike from now circulating Omicron lineages.

## MATERIALS AND METHODS

### Population and sampling

Immunocompromised individuals diagnosed with vasculitis were included in this study through the prospective observational “Covid-19 antibody responses in immunocompromised patient” Cohort (REC 20/EM/0180), examining SARS-CoV-2 antibody responses in immunocompromised individuals and the “VAccine Responses to understand ImmunE Dysfunction: VARIED study” (REC 24/PR/0097). Participants were recruited through the Department of Nephrology at Cambridge University Hospitals NHS Foundation Trust in the United Kingdom. Consent was provided from individuals partaking in these studies to contribute anonymized biological samples, prior vaccination and postvaccination, and clinical data. Ethical approval (REC 20/EM/0180) was provided by Leicester Central Research Ethics Committee on 9 July 2020 and by the Proportionate Review Sub-committee (REC 24/PR/0097) on 13 March 2024.

Immunocompetent participants were recruited through the NBR118 Study at the Cambridge NIHR BioResource Centre. Individuals consented to providing postvaccination biological samples as well as relevant clinical data. The study was approved by the East of England – Cambridge Central Research Ethics Committee (17/EE/0025) on 28 April 2020.

### Study design

Individuals diagnosed with vasculitis with a history of rituximab (+RTX) within the past 5 years were identified through the immunocompromised cohort. To investigate the potential impact of rituximab, we compared neutralizing titers between individuals diagnosed with vasculitis, distinguishing between those with and without a history of rituximab (±RTX), relative to immunocompetent individuals identified through the NBR118 Study (Fig. 1). In addition, we examined relative non-neutralizing binding antibody titers in individuals with vasculitis±RTX. All participants received a minimum of four WT vaccinations and were further stratified by primary vaccination type having received either two doses of BNT162b2 vaccine (Pfizer-BioNTech) (mRNA) or adenovirus-based AZD1222/ChAdOx1 nCoV-19 (AstraZeneca-Oxford) (AZN) vaccine, followed by two additional WT mRNA vaccines as part of routine clinical care.

### Cell culture and maintenance

Human embryonic kidney (HEK) 293T CRL-3216 cells containing the SV40 T-antigen were used as pseudotyped virus producer cells. HeLa-ACE2 cells stably transduced with angiotensin converting enzyme–2 (ACE2) receptor were used as target cells to measure serum neutralizing antibody titers against pseudotyped virus entry. All cells were maintained at 37°C in Dulbecco’s modified Eagle’s medium (DMEM) supplemented with glutamine, 10% fetal calf serum, 1% penicillin (100 ml/U), and streptomycin (0.1 mg/ml).

### SARS-CoV-2 pseudotyped virus production and neutralization assays

Viral entry of SARS-CoV-2 WT and Omicron-descendent variants in the presence of host neutralizing antibodies was evaluated using lentivirus-based pseudotyped viruses expressing luciferase, a method that mimics replication-competent infectious SARS-CoV-2 (*52*) virus but does not require the same biosafety laboratory standards. These methods have previously been described elsewhere (*53*–*56*). Briefly, spike pseudotyped viruses were produced by co-transfection of HEK 293T cells using 11 μl of Fugene HD Transfection Reagent (Promega) with 1 μg of an HIV-1 Gag-Pol-Tat-Rev packaging vector (p8.91), 1.5 μg of a firefly luciferase reporter gene construct with an HIV-1 packaging signal (pCSFLW), and 1 μg of a SARS-CoV-2 spike expression plasmid encoding a variant of interest (pcDNA3.1). The pcDNA3.1 plasmids contained codon-optimized reading frames encoding the SARS-CoV-2 spike of interests and a 19–amino acid deletion (Δ19) referencing an endoplasmic reticulum retention signal at the C termini, allowing for improved expression of spike proteins on pseudotype particles (*57*). HEK 293T cells were incubated at 37°C with 10% CO_2_, and viral supernatant was harvested 48 and 72 hours after transfection, passed through 0.45-μm filter, and stored at −80°C. Infectious dose of pseudotyped virus constructs was determined using the Bright-Glo Luciferase Assay System (Promega) and a GloMax Navigator Microplate Luminometer (Promega).

Collected sera were thawed from −80°C storage, heat-inactivated for 1 hour at 55°C, and diluted 1:10 using DMEM supplemented with glutamine, 10% fetal calf serum, 1% penicillin (100 ml/U), and streptomycin (0.1 mg/ml). Diluted sera were serially diluted alongside pseudotyped virus-only and cell-only control columns in 96-well white bottom plates and incubated with pseudotyped viruses for 1 hour at 37°C with 5% CO_2_ before the addition of HeLa-ACE2 target cells. Plates were subsequently incubated for 48 hours at 37°C with 5% CO_2_. Luminescence was measured using the Bright-Glo Luciferase Assay System (Promega) and a GloMax Navigator Microplate Luminometer (Promega), and data were normalized relative to pseudotyped virus-only and cell-only controls. All neutralizing assays were repeated in two sets of independent experiments, each comprising two technical replicates for each condition.

### ADCC response through Fc𝛄RIIIa (CD16) signaling assay

The ability of antibodies to cross-link with spike-expressing cells and signal through FcγRIIIa (CD16) was measured to represent ADCC (*58*, *59*). To perform the assay, HEK 293T cells were plated at a cell density of 2 × 10^−4^ cells per well in a white tissue culture–treated 96-well plate and incubated for 4 hours at 5% CO_2_ and 37°C. Following incubation, cells were transiently transfected with 5 mg (50 ng of DNA per well) of SARS-CoV-2 spike plasmid using Fugene HD Transfection Reagent (Promega) and incubated at 5% CO_2_ and 37°C for 48 hours. On the day of performing the assay, the medium on the transfected cells was replaced with 25 μl of assay medium (1× RPMI 1640 supplemented with 4% low-IgG fetal bovine serum) and placed in incubator at 5% CO_2_ and 37°C. After replacing the medium, in a separate 96-well plate, heat-inactivated sera (1:90 final dilution) or monoclonal antibody (30 ng/μl) was diluted in the previously described RPMI 1640 assay media (Gibco) and transferred to the appropriate wells in the tissue culture–treated 96-well plate with transfected cells for 1 hour at 5% CO_2_ and 37°C. Each sample was repeated in two sets of independent experiments, each comprising two technical replicates for each condition. Following incubation, Jurkat-Lucia NFAT-CD16 cells (Promega) were added at a concentration of 75,000 cells per well and incubated for 18 hours at 5% CO_2_ and 37°C. Fifty microliters of Bright-Glo (Promega) was added to each well, and luminescence was read using the Bright-Glo Luciferase Assay System (Promega) and a GloMax Navigator Microplate Luminometer (Promega). Normalized relative light units (RLU) were calculated by subtracting the “medium-only” and “cell-only” background. Sotrovimab monoclonal antibody from GlaxoSmithKline served as a positive control, and two seropositive plasma controls, one high ADCC reporter and one low ADCC reporter, were included on each plate. Plates were normalized per positive and negative controls. Each plate contained sotrovimab and two heat-inactivated SARS-CoV-2 seropositive plasma controls: untransfected cells and transfected cells as positive controls, and a “cell-only” and “medium-only” condition as negative controls.

### Virus variants

SARS-CoV-2 spike–expressing plasmids for generation of pseudotyped virus were provided by T. P. Peacock and W. Barclay, Imperial College London, London, UK, through the Genotype-to-Phenotype National Virology Consortium (G2P-UK). All spike variants were produced using pcDNA3.1 plasmids that were codon optimized to human codon usage and contained Δ19 region at the C termini of the spike gene. The WT isolate (Wuhan-Hu-1) included mutation D614G. The BA.1 Omicron variant included mutations A67V, Δ69-70, T95I, G142D/Δ143-145, Δ211/L212I, ins214EPE, G339D, S371L, S373P, S375F, K417N, N440K, G446S, S477N, T478K, E484A, Q493R, G496S, Q498R, N501Y, Y505H, T547K, D614G, H655Y, N679K, P681H, N764K, D796Y, N856K, Q954H, N969K, and L981F. The BA.2 Omicron variant included mutations T19I, L24S/Δ25-27, G142D, V213G, G339D, S371F, S373P, S375F, T376A, D405N, R408S, K417N, N440K, S477N, T478K, E484A, Q493R, Q498R, N501Y, Y505H, D614G, H655Y, N679K, P681H, N764K, D796Y, Q954H, and N969K. The BA.4/5 variant included mutations T19I, L24S/Δ25-27, Δ69-70, G142D, V213G, G339D, S371F, S373P, S375F, T376A, D405N, R408S, K417N, N440K, L452R, S477N, T478K, E484A, F486V, Q498R, N501Y, Y505H, D614G, H655Y, N679K, P681H, N764K, D796Y, Q954H, and N969K. The XBB variant included mutations T19I, L24S/Δ25-27, V83A, G142D, Δ145, H146Q, Q183E, V213E, G339H, R346T, L368I, S371F, S373P, S375F, T376A, D405N, R408S, K417N, N440K, V445P, G446S, N460K, S477N, T478K, E484A, F486S, F490S, Q493R, Q498R, N501Y, Y505H, D614G, H655Y, N679K, P681H, N764K, D796Y, Q954H, and N969K.

### Statistical analysis

The ID_50_ was calculated using GraphPad Prism version 9.5.1. A value of ID_50_ > 40 was considered positive as the assay limits of detection range between 40 and 87,480, and values are constrained to these limits. ID_50_ values were summarized using GMT with geometric SD within each group. The maximal normalized titer post 1:90 dilution was calculated to assess ADCC response. Each sample varied in limit of detection range dependent on the plate-specific positive and negative controls.

Data analysis was performed using statistical software R v4.3.0 (*60*, *61*) with packages readr (v2.1.4), tidyr (v1.3.0), tibble (v3.2.1), ggplot2 (v3.4.2), tidyverse (v2.0.0), splitstackshape (v1.4.8), dplyr (v1.1.2), readxl (v1.4.2), lubridate (v1.9.2), ggsci (v3.0.0), writexl (v1.4.2), and viridis (v0.6.3).

### IgG binding antibodies

IgG binding antibodies against the SARS-CoV-2 nucleocapsid (N) protein, spike (S) protein, and receptor binding domain were identified by a multiplex luminex–based assay, as previously described (*53*, *62*). Briefly, IgG binding titers against the S-protein were identified using a closed, pre-fusion, thermostable S-protein timer with a locked conformation due to engineered disulfide bonds. Specific antibody binding was reported as mean fluorescent intensity (MFI), and limits for negativity and positivity were identified using pre-pandemic and post–SARS-CoV-2 infection convalescent samples, respectively. Previous SARS-CoV-2 infection was defined as an anti-N measurement above 6104 MFI. Positive markers for vaccine-induced binding IgG antibodies were defined as an anti-S MFI above 1896.

### Matching

Intragroup comparisons occurred post-matching on the basis of individual data. To control for possible confounding effects of age and sex at time of initial recruitment, vasculitis+RTX individuals within the stratified AZN and mRNA subgroups were matched using a 1:1 nearest neighbor-matching method dependent on a propensity score to achieve covariate balance between groups (fig. S7A). Subsequently, coarsened exact matching (*63*), where covariates are organized into bins to form subclasses, occurred between the vasculitis (±RTX) and immunocompetent groups on the basis of primary vaccination type (fig. S7, B to E) where overall sampling weights were equivalent between groups. Inter-variance ratios between vasculitis (±RTX) and control groups were confirmed to be within a range indicative of covariance balance (table S1) and did not require further removal of samples. Matching occurred using R v4.3.0 with package MatchIt (v4.5.4) (*64*), and methods “nearest” for nearest neighbor matching and “cem” for coarsened exact matching were used.

### Generalized linear mixed model

A generalized linear mixed model was used with a gamma distribution and a log link function for modeling non-normal neutralizing antibody titers against fixed effects of (i) IgG binding antibody titers, (ii) group (±RTX), and (iii) interactive group with IgG binding titers. Individuals were treated as random effects to account for nonindependence. Continuous variables were transformed and rescaled to a near normal range using Box-Cox function in Caret package (v 6.0.94). The multiplicative effect of each variable was determined post-dose 3 and post-dose 4. Analysis occurred using R v4.3.0 (*61*) with distribution determined using package fitdistrplus (v 1.1.11) (*65*) and model building using lme4 (v1.1.34) (*66*) with the glmer function.

### Antigenic map

The antigenic map was constructed using antigenic cartography (*67*) with the “Racmacs” (*68*) package in R (*61*) (v4.2.1) using neutralizing data using 1 week postimmunization mouse serum provided by Y. Cao. As previously described elsewhere (*69*), the table of neutralizing titers is converted into a distance table by calculating the log_2_fold change from the maximum titer for each serum to all other titers per serum. Coordinates for each serum and variant pair are then optimized such that their Euclidean distance in the map matches their table distance. A detailed description of the algorithm is given by Smith *et al.* (*67*) and the reference page of the Racmacs package.

### Antibody landscape

Antibody landscapes were constructed using the “ablandscapes” package (*70*). For each serum group, single-cone landscapes were fit to each individual serum, and the GMT landscape was calculated by taking the average of the landscape for the individual sera in that group. However, as many individuals have a large number of titers below the limit of detection (sometimes all titers) and, therefore, cannot provide information about the shape of the antibody landscape, landscapes were fit using a three-step process:

i) The overall slope of the landscape was estimated using titration from individuals with at least three detectable titers. The height and coordinates of the peak of the landscape was also estimated for these individuals.

ii) For the remaining individuals (with fewer than three detectable titers), the landscape parameters cannot be reliably estimated. Therefore, the coordinates of the peak of the landscape were randomly sampled from the estimates for the individuals in (i). The height of the landscape was then estimated with the slope fixed to the value estimated in (i) and with landscape peak coordinates fixed to the randomly sampled value.

iii) The summary GMT landscape was produced by taking the mean of the all the individual landscapes from (i) and (ii).

### Bayesian titer estimation

In data containing a large number of non-detectable titers, the commonly used method of treating non-detectable titers as 0.5× the lower limit of detection is likely to overestimate the true GMT. To estimate GMTs, we, therefore, use the “gmt_me” function from the “titertools” package (*71*), which uses a Bayesian method to estimate GMTs while accounting for thresholding of titers and biases in serum and antigen reactivity. Details of the method have been described previously (*72*).

## Supplementary Materials

### This PDF file includes:

Figs. S1 to S7 The CITIID-NIHR BioResource COVID-19 Collaboration authorship list
