# Supplementary material for: Humoral responses to SARS-CoV-2 vaccine in vasculitis-related immune suppression
Source: Sci Adv. 2025 Feb 12;11(7):eadq3342. doi: 10.1126/sciadv.adq3342 (PMC11817922; doi:10.1126/sciadv.adq3342)
Supplement: Supplementary file 1 — Figs. S1 to S7 The CITIID-NIHR BioResource COVID-19 Collaboration authorship list [file sciadv.adq3342_sm.pdf]

Science Advances
AAAS

Supplementary Materials for

Humoral responses to SARS-CoV-2 vaccine in vasculitis-related immune suppression

Kimia Kamelian *et al.*

Corresponding author: Ravindra K. Gupta, rkg20@cam.ac.uk

Sci. Adv. **11**, eadq3342 (2025)
DOI: 10.1126/sciadv.adq3342

This PDF file includes:

Figs. S1 to S7
The CITIID-NIHR BioResource COVID-19 Collaboration authorship list

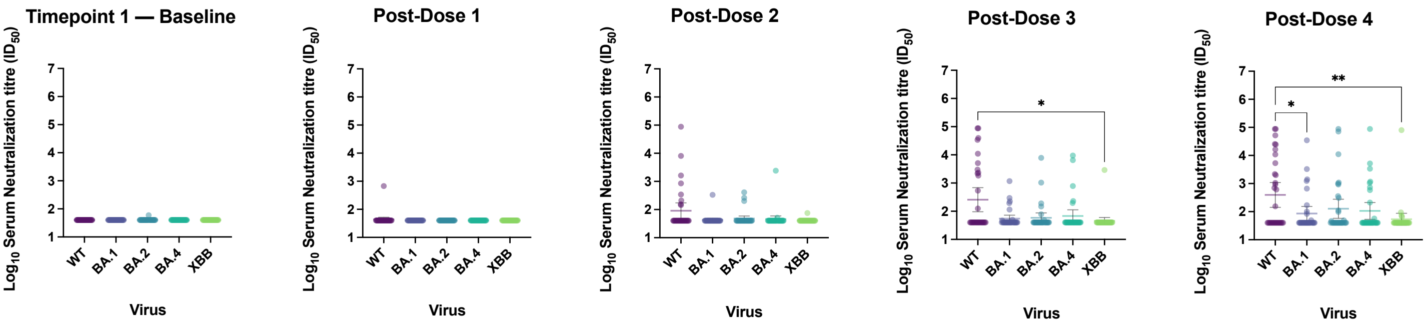

Fig. S1. Neutralization is less likely to be achieved with more recent antigenically distinct variants in longitudinal cohort.

Post-dose 2, post-dose 3, and post-dose 4 vaccination showed significant differences in neutralizing titres (Freidman test; post-dose 2, p<0.0001; post-dose 3, p<0.0001, post-dose 4, p<0.0001). Pairwise post-hoc comparisons using Dunn’s multiple comparison's test indicated significant differences between WT and XBB response post-dose 3 (adjusted p=0.03) and post-dose 4 vaccinations (adjusted p=0.004), as well as WT and BA.1 response post-dose 4 vaccination (adjusted p=0.03).

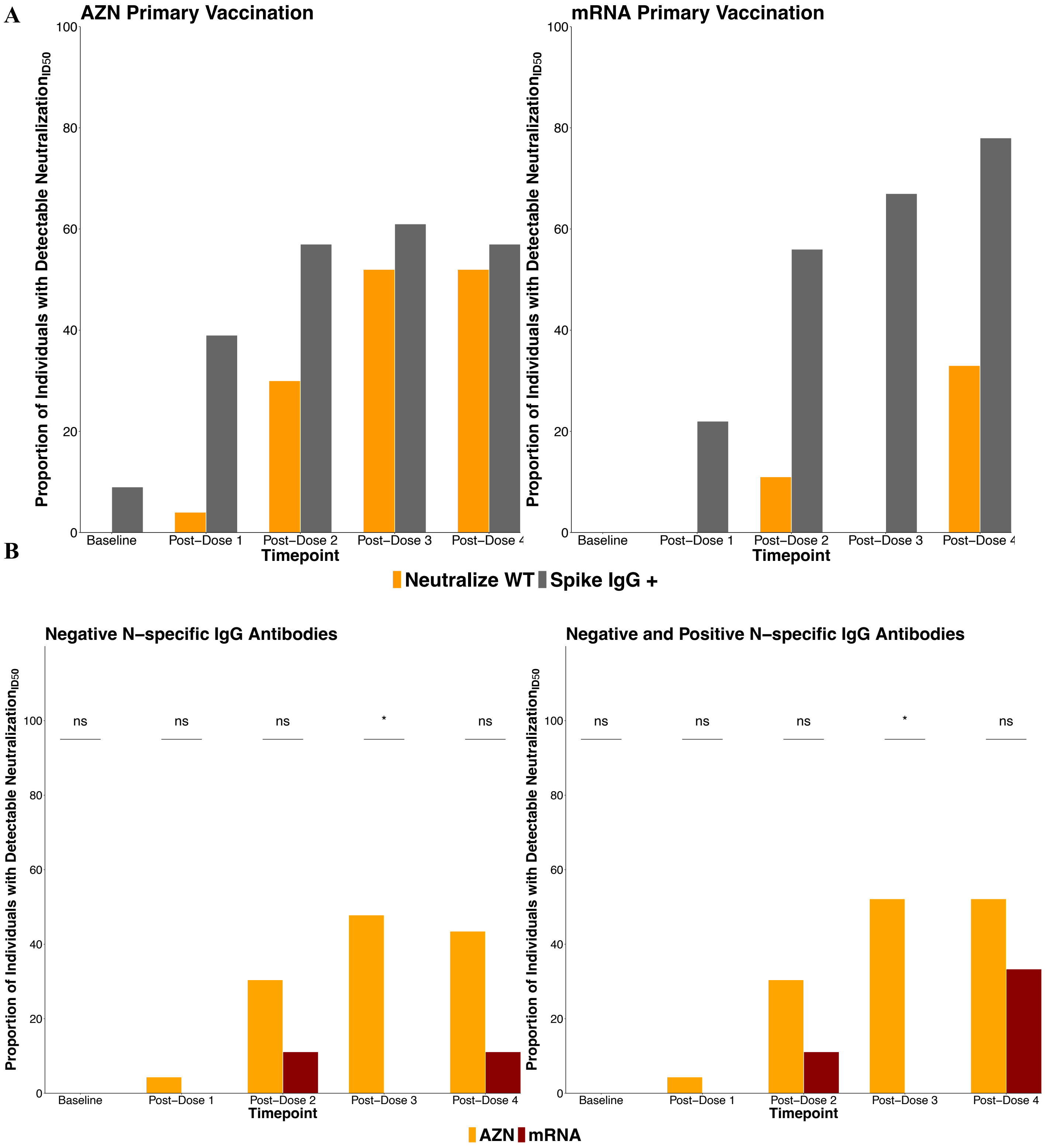

Fig. S2. Serum neutralizing responses and binding IgG titers in longitudinal cohort.
A. The discordant neutralizing response is predominantly observed in mRNA primary vaccination group (n=23) versus AZN (n=9) **B.** Stratification of longitudinal responses by N-specific IgG antibody. No significant differences were observed between primary vaccination groups when stratified by N-specific IgG antibodies as determined by Fisher's exact test (p>0.05), except at post-dose 3 vaccination (negative N-specific IgG, p=0.01; negative and positive N-specific IgG, p=0.01).

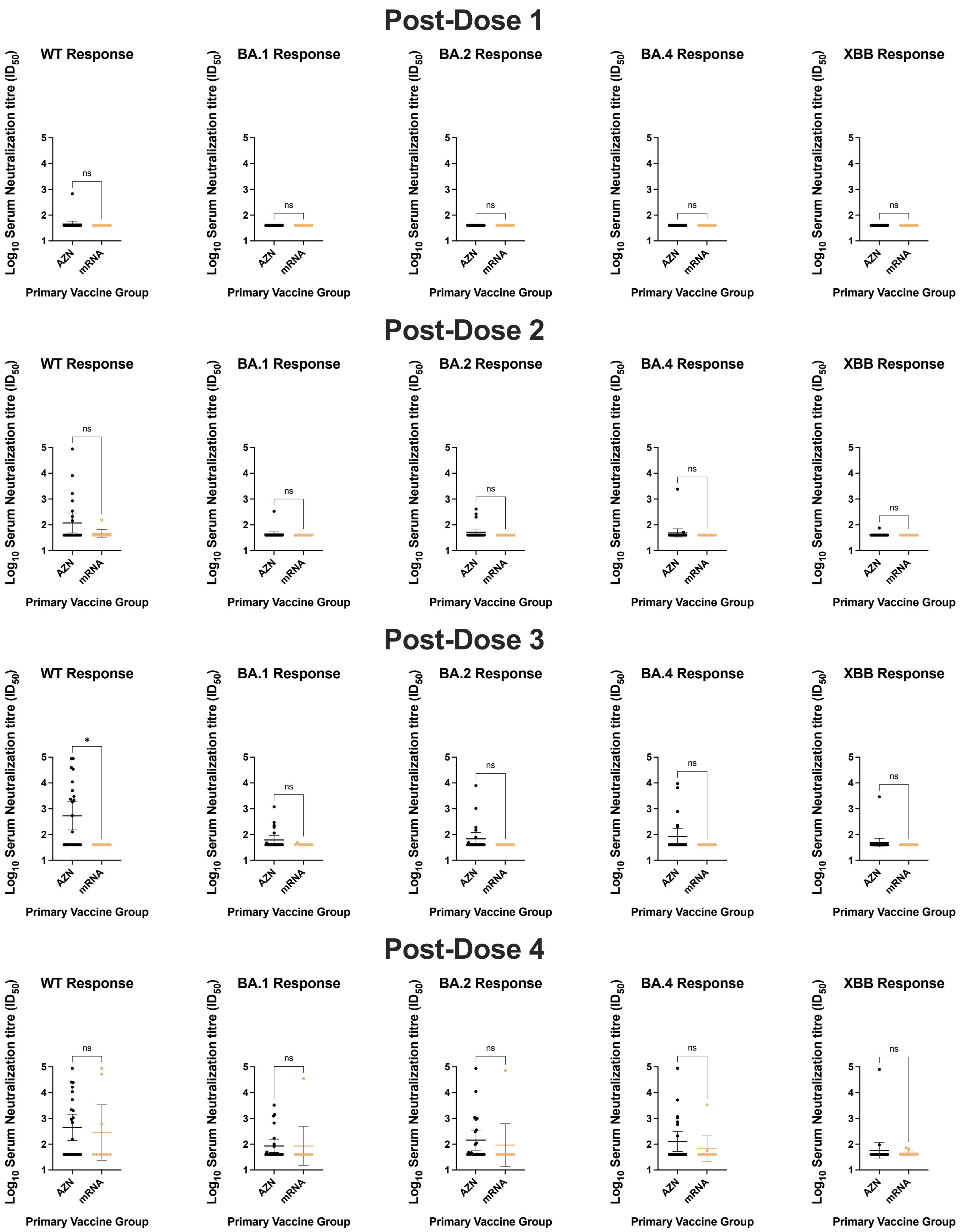

Fig. S3. Primary vaccination platform and neutralizing titres in longitudinal cohort. Neutralizing titres at post-dose 1, 2, 3, or 4 (Mann-Whitney test) for WT, BA.1, BA.2, BA.4, or XBB between AZN and mRNA primary vaccination groups.

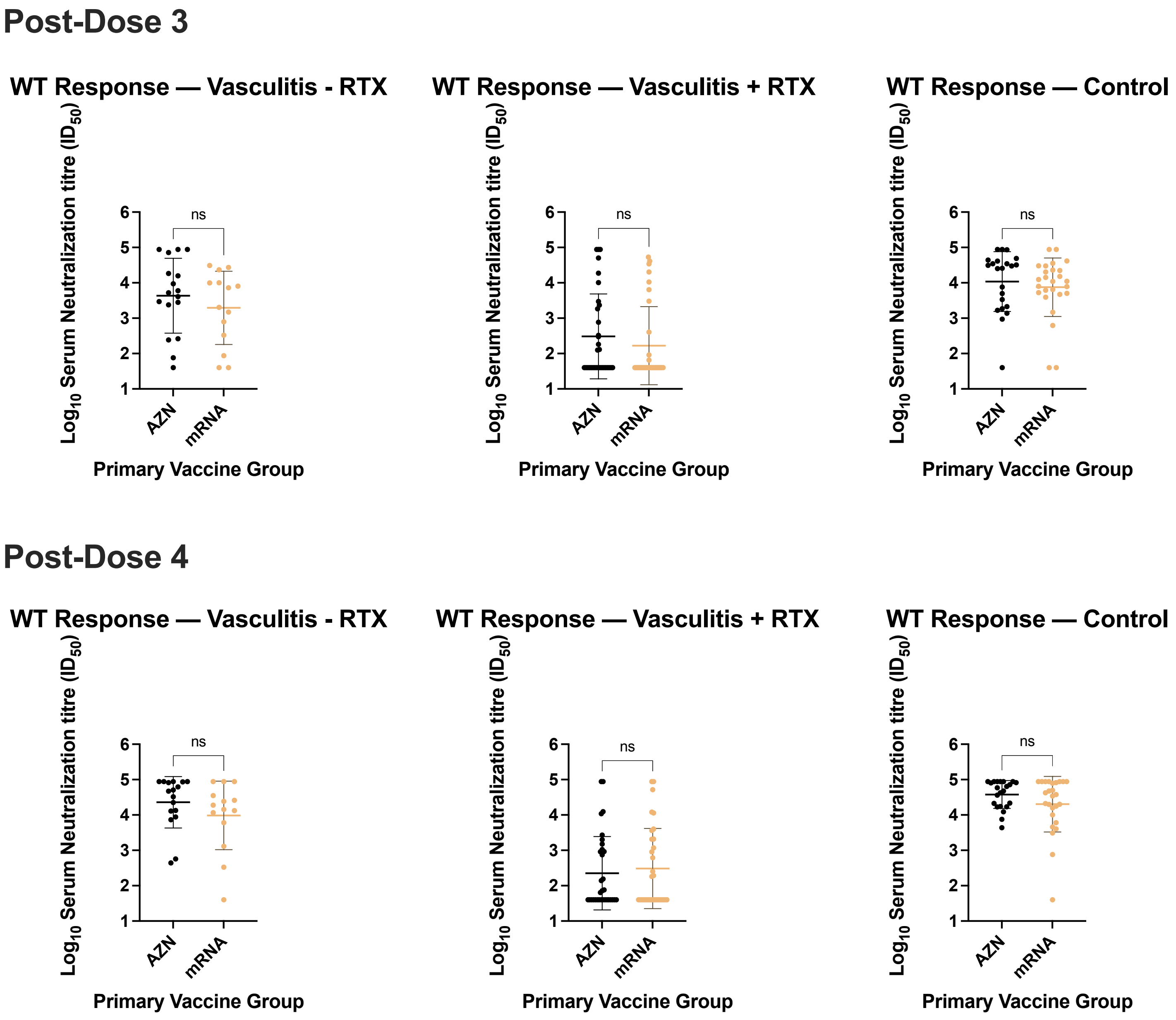

Fig. S4. Neutralizing antibody titres in case-control study stratified by primary vaccination platform. There is no statistical difference between WT neutralizing responses between primary vaccination groups post-dose 3 or post-dose 4 vaccination in vasculitis-RTX, vasculitis+RTX, or control group.

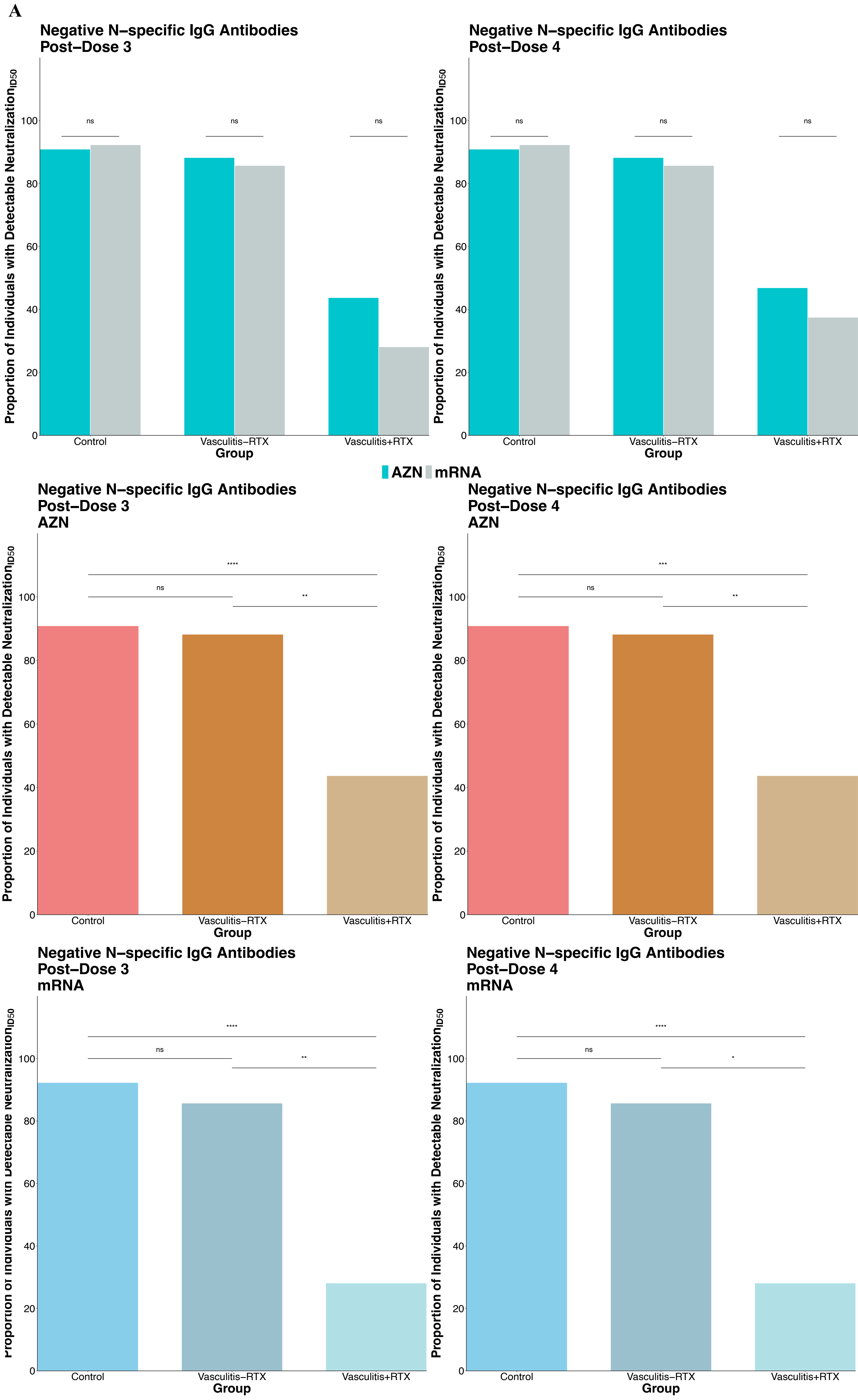

A
Negative N−specific IgG Antibodies Post−Dose 3
Negative N−specific IgG Antibodies Post−Dose 4
Proportion of Individuals with Detectable Neutralization$_{ID50}$
ns
Control
Vasculitis−RTX
Vasculitis+RTX
Group
AZN
mRNA
Negative N−specific IgG Antibodies Post−Dose 3 AZN
Negative N−specific IgG Antibodies Post−Dose 4 AZN
Negative N−specific IgG Antibodies Post−Dose 3 mRNA
Negative N−specific IgG Antibodies Post−Dose 4 mRNA
****
***
**
*

B

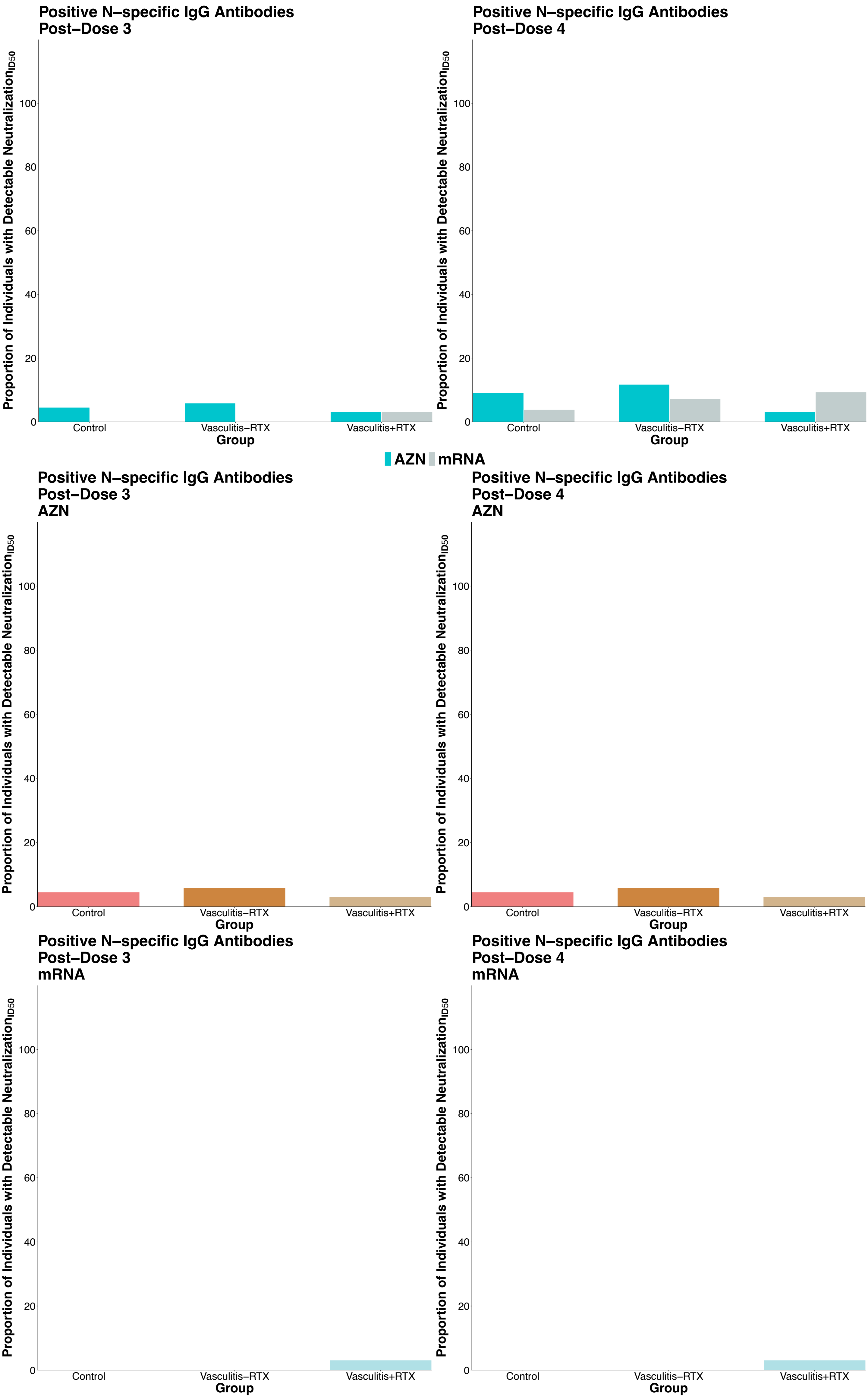
Positive N–specific IgG Antibodies
Post–Dose 3
Positive N–specific IgG Antibodies
Post–Dose 4
Proportion of Individuals with Detectable Neutralization$_{ID50}$
0
20
40
60
80
100
Control
Vasculitis–RTX
Vasculitis+RTX
Group
AZN
mRNA
Positive N–specific IgG Antibodies
Post–Dose 3
AZN
Positive N–specific IgG Antibodies
Post–Dose 4
AZN
Positive N–specific IgG Antibodies
Post–Dose 3
mRNA
Positive N–specific IgG Antibodies
Post–Dose 4
mRNA

C

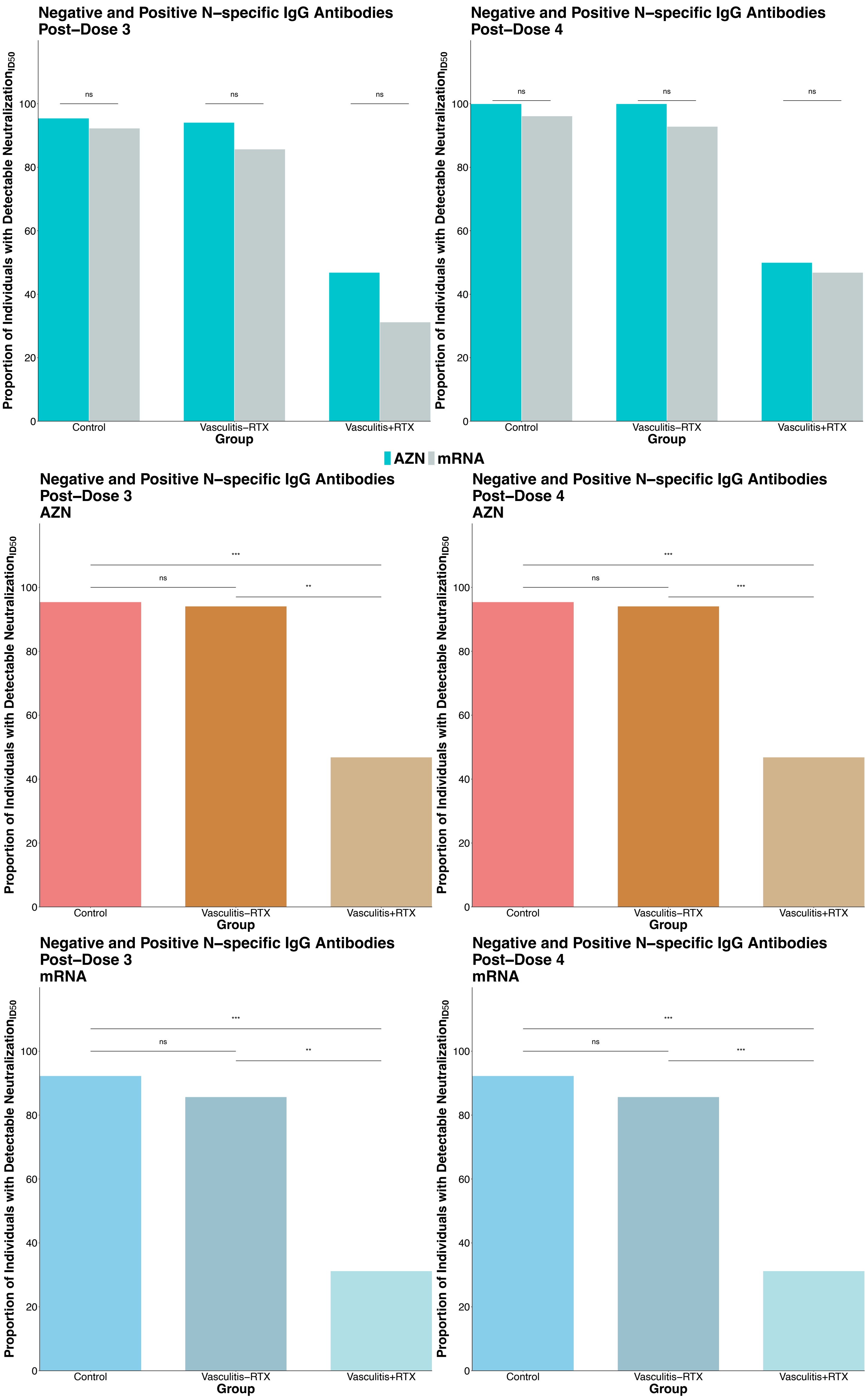

Negative and Positive N–specific IgG Antibodies Post–Dose 3
Negative and Positive N–specific IgG Antibodies Post–Dose 4
Proportion of Individuals with Detectable Neutralization$_{ID50}$
ns
ns
ns
Control
Vasculitis–RTX
Vasculitis+RTX
Group
AZN
mRNA
Negative and Positive N–specific IgG Antibodies Post–Dose 3 AZN
Negative and Positive N–specific IgG Antibodies Post–Dose 4 AZN
***
**
Negative and Positive N–specific IgG Antibodies Post–Dose 3 mRNA
Negative and Positive N–specific IgG Antibodies Post–Dose 4 mRNA
0
20
40
60
80
100

Fig. S5. Natural infection increases proportion of individuals with detectable neutralizing titers, but immunocompromised status and rituximab treatment severely impair neutralizing responses.

Stratification of serum neutralization by N-specific IgG responses between primary vaccination type within immunocompetent, vasculitis-RTX, and vasculitis+RTX groups as determined by Fisher's exact test.

(ns: not significant; *: $p<0.05$; ** : $p < 0.01$; *** : $p < 0.001$, **** : $p < 0.0001$).

A. Proportion of individuals with negative N-specific IgG antibodies representing absence of prior infection were identified post-dose 3 and post-dose 4. Difference between vaccination type within each group (immunocompetent controls, vasculitis-RTX, vasculitis+RTX) were not statistically significant.

B. Proportion of individuals with positive N-specific IgG antibodies representing prior infection were identified post-dose 3 and post-dose 4. Test of statistical significance could not occur due to small sample size.

C. Proportion of individuals with negative and positive N-specific IgG antibodies were identified post-dose 3 and post-dose 4. Difference between vaccination type were not statistically significant. Difference between vaccination type within each group (immunocompetent controls, vasculitis-RTX, vasculitis+RTX) were not statistically significant.

A

Post-Dose 3

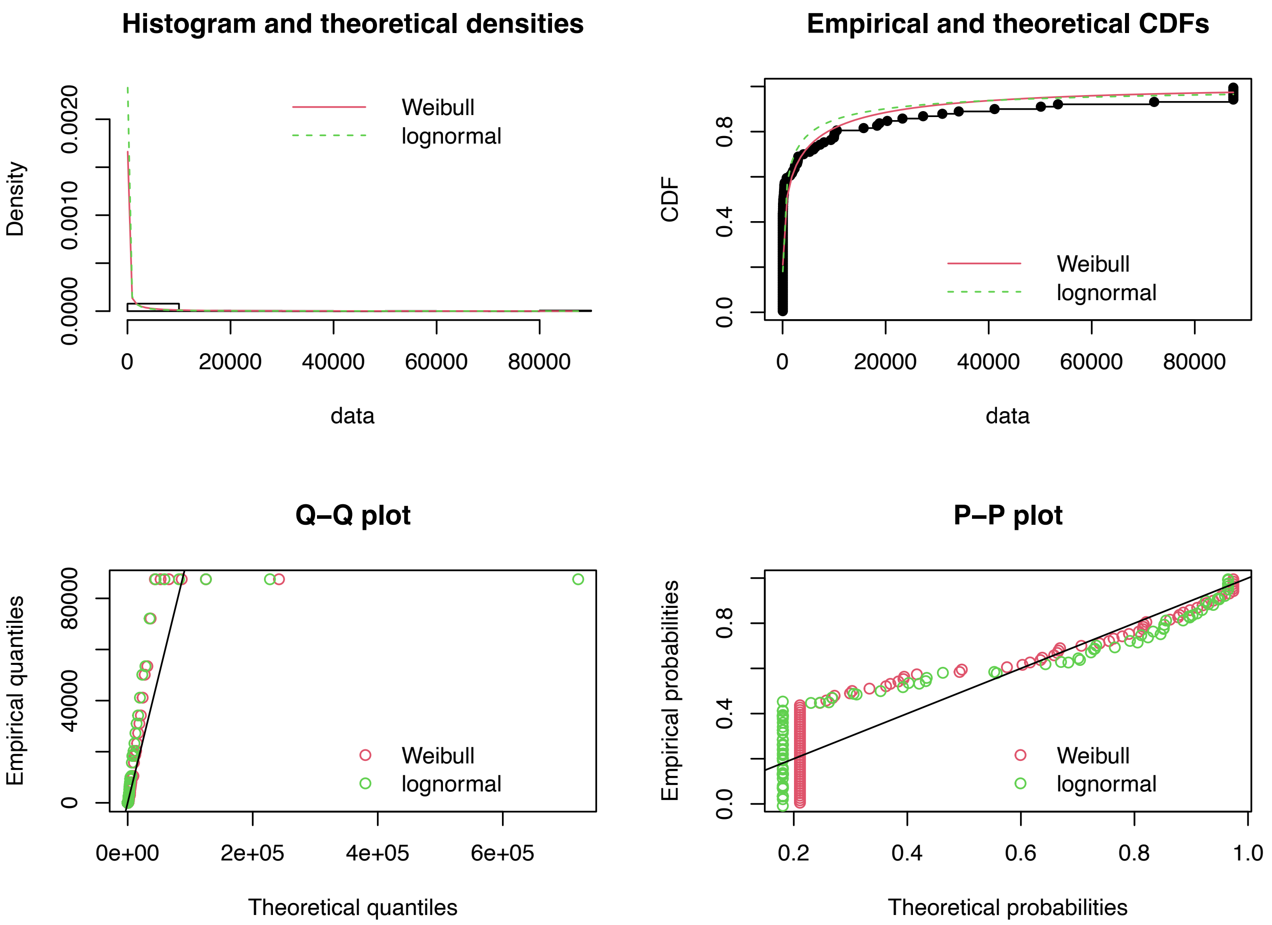

Post-Dose 4

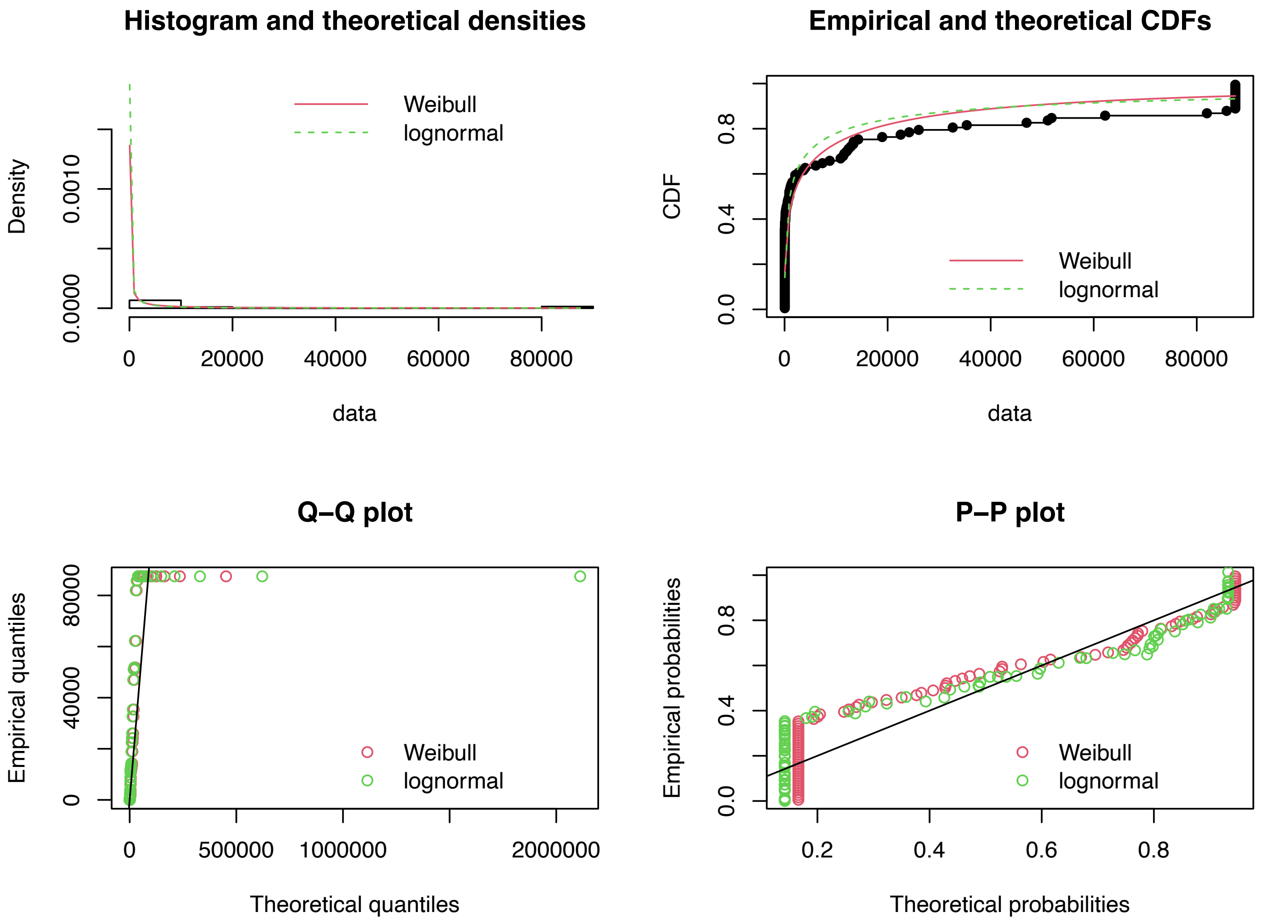

B

Post-Dose 3

```
Generalized linear mixed model fit by maximum likelihood (Laplace Approximation) ['glmerMod']
 Family: Gamma  ( log )
Formula: IC50_4 ~ group_factor + predicted_value_s + group_factor * predicted_value_s +      (1 | studyid_factor)
   Data: df_T3
Control: glmerControl(optimizer = "Nelder_Mead")

     AIC      BIC   logLik deviance df.resid
  -363.0   -347.7    187.5   -375.0       89

Scaled residuals:
       Min         1Q     Median         3Q        Max
-4.922e-05 -2.552e-05 -1.462e-05  7.513e-06  7.616e-05

Random effects:
 Groups         Name        Variance  Std.Dev.
 studyid_factor (Intercept) 5.382e+00 2.320e+00
 Residual                   3.996e-09 6.321e-05
Number of obs: 95, groups:  studyid_factor, 95

Fixed effects:
                                       Estimate Std. Error t value Pr(>|z|)
(Intercept)                           -2.913613   3.110641  -0.937  0.34893
group_factorVas-rit                    7.727701   3.147198   2.455  0.01407 *
predicted_value_s                      0.014225   0.004011   3.547  0.00039 ***
group_factorVas-rit:predicted_value_s -0.010574   0.004107  -2.575  0.01002 *
---
Signif. codes:  0 '***' 0.001 '**' 0.01 '*' 0.05 '.' 0.1 ' ' 1

Correlation of Fixed Effects:
            (Intr) grp_V- prdc__
grp_fctrVs- -0.988
prdctd_vl_s -0.991  0.979
grp_fcV-:__  0.968 -0.983 -0.977
optimizer (Nelder_Mead) convergence code: 0 (OK)
Gradient contains NAs
```

Post-Dose 4

```
Generalized linear mixed model fit by maximum likelihood (Laplace Approximation) ['glmerMod']
 Family: Gamma  ( log )
Formula: IC50_4 ~ group_factor + predicted_value_s + group_factor * predicted_value_s +      (1 | studyid_factor)
   Data: df_T4
Control: glmerControl(optimizer = "Nelder_Mead")

     AIC      BIC   logLik deviance df.resid
  -240.5   -225.2    126.3   -252.5       89

Scaled residuals:
       Min         1Q     Median         3Q        Max
-6.527e-05 -1.640e-05 -1.719e-06  2.450e-05  9.975e-05

Random effects:
 Groups         Name        Variance  Std.Dev.
 studyid_factor (Intercept) 3.245e+00 1.801e+00
 Residual                   3.947e-09 6.282e-05
Number of obs: 95, groups:  studyid_factor, 95

Fixed effects:
                                       Estimate Std. Error t value Pr(>|z|)
(Intercept)                           -2.391383   2.802320  -0.853  0.39346
group_factorVas-rit                    6.213500   2.828145   2.197  0.02802 *
predicted_value_s                      0.014201   0.003519   4.035 5.45e-05 ***
group_factorVas-rit:predicted_value_s -0.010006   0.003587  -2.789  0.00528 **
---
Signif. codes:  0 '***' 0.001 '**' 0.01 '*' 0.05 '.' 0.1 ' ' 1

Correlation of Fixed Effects:
            (Intr) grp_V- prdc__
grp_fctrVs- -0.991
prdctd_vl_s -0.993  0.984
grp_fcV-:__  0.975 -0.987 -0.981
optimizer (Nelder_Mead) convergence code: 0 (OK)
Gradient contains NAs
```

Fig. S6. Generalized linear mixed model.
A. Lognormal distribution best reflected range of ID_{50} values post-dose 3 and post-dose 4 vaccination for vasculitis (±RTX) individuals suggesting gamma-like distribution with log link function.
B. Generalized linear mixed model is fit by maximum likelihood and optimized using the Nelder-Mead method. Fixed variables included scaled IgG binding titres, group (±RTX), and interactive group with scaled IgG binding titres. Individuals were treated as random effects to account for non-independence.

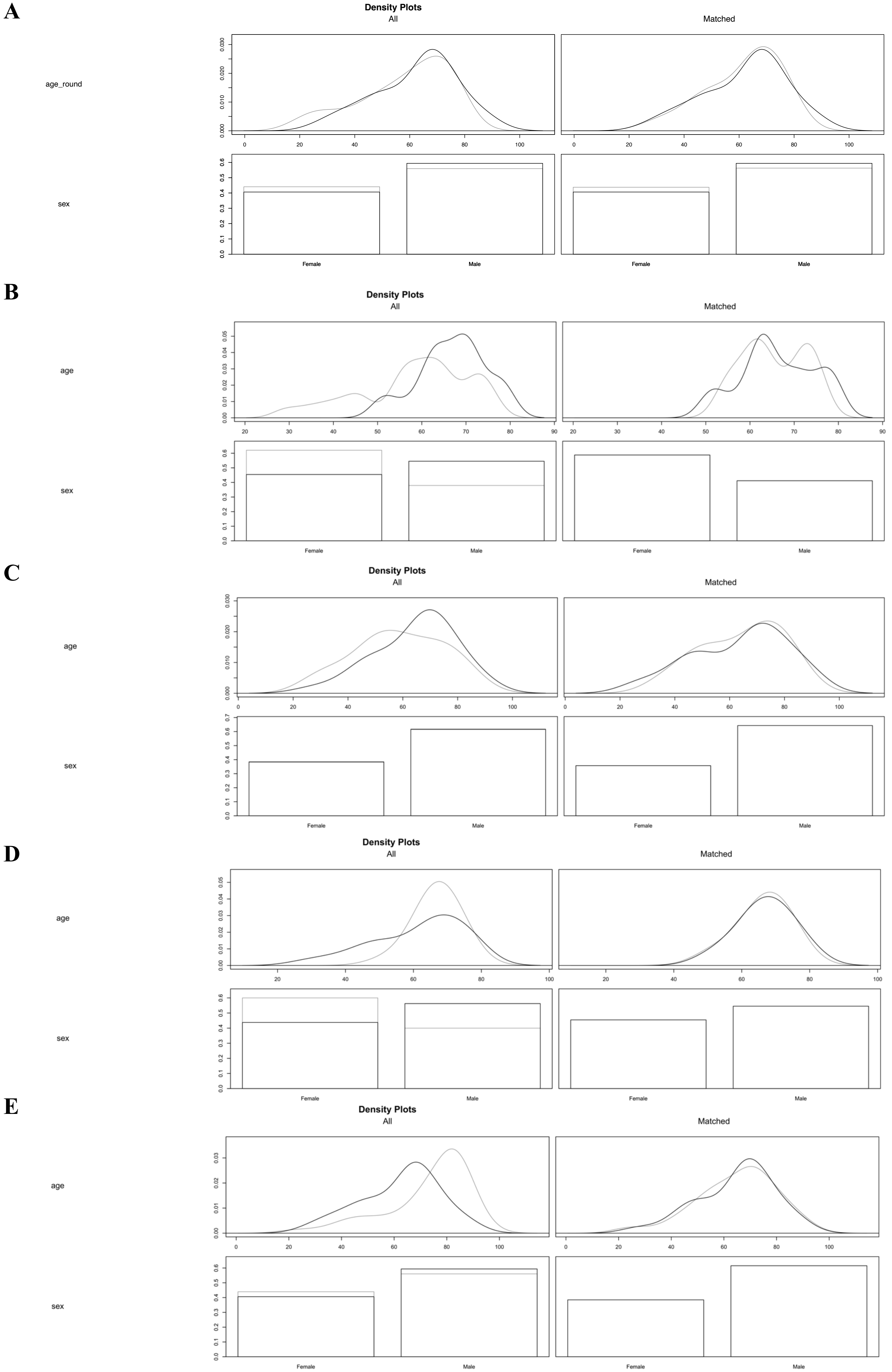

Fig. S7. Inter- and Intra-matching of vasculitis and control samples.

A. Density plots indicating prior- and post-matching means of propensity scores between AZN and mRNA primary vaccines within individuals diagnosed with vasculitis and treated with rituximab (RTX) **B.** Density plots indicating prior- and post-matching means of propensity scores for between individuals diagnosed with vasculitis, receiving AZN primary vaccination, and treated with and without RTX **C.** Density plots indicating prior- and post-matching means of propensity scores for between individuals diagnosed with vasculitis, receiving mRNA primary vaccination, and treated with and without RTX **D.** Density plots indicating prior- and post-matching means of propensity scores for between individuals diagnosed with vasculitis and treated with RTX against controls, receiving AZN primary vaccination **E.** Density plots indicating prior- and post-matching means of propensity scores for between individuals diagnosed with vasculitis and treated with RTX against controls, receiving mRNA primary vaccination.

The CITIID-NIHR BioResource COVID-19 Collaboration Authorship Banner

Stephen Baker[2,6], John Bradley[1,3,6,11,15] , Patrick Chinnery[3,23,24], Daniel Cooper[11, 25], Gordon Dougan[2,6], Ian Goodfellow[7], Ravindra Gupta[2,6,13,16], Nathalie Kingston[3,4], Paul J. Lehner[2,6,13], Paul A. Lyons[2,6], Nicholas J. Matheson[2,6,13,33], Caroline Saunders[9], Kenneth G. C. Smith[2,6], Charlotte Summers[6,12,26], James Thaventhiran[19], M. Estee Torok [6,13,14], Mark R. Toshner[6,8,26], Michael P. Weekes[2,6,13,34], Gisele Alvio[9], Sharon Baker[9], Areti Bermperi[9], Karen Brookes[9], Ashlea Bucke, Jo Calder, Laura Canna, Cherry Crucusio, Isabel Cruz[9], Ranalie de Jesus[9], Katie Dempsey[9], Giovanni Di Stephano[9], Jason Domingo[9], Anne Elmer[9], Julie Harris, Sarah Hewitt, Heather Jones[9], Sherly Jose[9], Jane Kennet, Yvonne King, , Jenny Kourampa[9], Emily Li, Caroline McMahon[9], Anne Meadows, Vivien Mendoza[9], Criona O'Brien, Charmain Ocaya[9], Ciro Pasquale[9], Marlyn Perales[9], Jane Price, Rebecca Rastall, Carla Ribeiro[9], Jane Rowlands, Valentina Ruffolo, Hugo Tordesillas, Phoebe Vargas[9], Bensi Vergese[9], Laura Watson[9], Jieniean Worsley[9], Julie-Ann Zerrudo[9], Laura Bergamashi[2,6], Ariana Betancourt, Georgie Bower, Ben Bullman, Chiara Cossetti, Aloka De Sa, Benjamin J. Dunmore, Maddie Epping, Stuart Fawke, Stefan Gräf [3,6], Richard Grenfell, Andrew Hinch, Josh Hodgson, Christopher Huang, Oisin Huhn, Kelvin Hunter[2,6], Isobel Jarvis, Emma Jones, Maša Josipović, Ekaterina Legchenko, Daniel Lewis, Joe Marsden, Jennifer Martin, Federica Mescia[2,6], Ciara O'Donnell, Ommar Omarjee, Marianne Perera, Linda Pointon, Nicole Pond, Nathan Richoz, Nika Romashova, Natalia Savoinykh, Rahul Sharma, Joy Shih, Mateusz Strezlecki, Rachel Sutcliffe, Tobias Tilly, Zhen Tong, Carmen Treacy, Lori Turner, Jennifer Wood, Marta Wylot, John Allison[3,4], Heather Biggs[3,18], John R. Bradley[1,3,6,11,15], , Helen Butcher[3,5], Daniela Caputo[3,5], Matt Chandler[3,5], Patrick Chinnery[3,23,24], Debbie Clapham-Riley[3,5], Eleanor Dewhurst[3,5], Christian Fernandez[3,] Anita Furlong[3,5], Barbara Graves[3,5], Jennifer Gray[3,5], , Sabine Hein[3,5], Tasmin Ivers[3,5], Emma Le Gresley[3,5], Rachel Linger[3,5], Mary Kasanicki[3,11], Rebecca King, Nathalie Kingston[3,4], Sarah Meloy[3,5], Alexei Moulton[3,5], Francesca Muldoon[3,5], Nigel Ovington[3,4], Sofia Papadia[3,5], Christopher J. Penkett[3,4], Isabel Phelan[3,5], Venkatesh Ranganath[3,4], Roxana Paraschiv[3,4], Abigail Sage[3,5], Jennifer Sambrook[3,4], Ingrid Scholtes, Katherine Schon[3,17,18], Hannah Stark[3,5], Kathleen E. Stirrups[3,4], Paul Townsend[3,4], Neil Walker[3,4], Jennifer Webster[3,5], Mayurun Selvan, Petra, Polgarova[12],Sarah L. Caddy[2,6], Laura G. Caller[20,21], Yasmin Chaudhry[7], Martin D. Curran[22], Theresa Feltwell[6], Stewart Fuller[20], Iliana Georgana[7], Grant Hall[7], William L. Hamilton[6,13,14], Myra Hosmillo[7], Charlotte J. Houldcroft[6], Rhys Izuagbe[7], Aminu S. Jahun[7], Fahad A. Khokhar[2,6], Anna G. Kovalenko[7], Luke W. Meredith[7], Surendra Parmar[22], Malte L. Pinckert[7],

Anna Yakovleva[7], Emily C. Horner[19], Lucy Booth[19], Alexander Ferreira[19], Rebecca Boston[19], Robert Hughes[19], Juan Carlos Yam Puc[19], Nonantzin Beristain-Covarrubias[19], Maria Rust[19], Thevinya Gurugama[19], Lihinya Gurugama[19], Thomas Mulroney[19], Sarah Spencer[19], Zhaleh Hosseini[19], Kate Williamson[19].

[1]NIHR Cambridge Biomedical Research Centre, Cambridge Biomedical Campus, Cambridge, UK

[2]Cambridge Institute of Therapeutic Immunology and Infectious Disease (CITIID), Jeffrey Cheah Biomedical Centre, Cambridge Biomedical Campus, Cambridge, UK

[3]NIHR BioResource, Cambridge University Hospitals NHS Foundation Trust, Cambridge Biomedical Campus, Cambridge, UK

[4]Department of Haematology, School of Clinical Medicine, University of Cambridge, Cambridge Biomedical Campus, Cambridge, UK

[5]Department of Public Health and Primary Care, School of Clinical Medicine, University of Cambridge, Cambridge Biomedical Campus, Cambridge, UK

[6]Department of Medicine, School of Clinical Medicine, University of Cambridge, Cambridge Biomedical Campus, Cambridge, UK

[7]Division of Virology, Department of Pathology, University of Cambridge, Cambridge, UK

[8]Royal Papworth Hospital NHS Foundation Trust, Cambridge, UK

[9]Cambridge Clinical Research Centre, Addenbrooke's Hospital, Cambridge University Hospitals NHS Foundation Trust, Cambridge, UK

[10]Intensive Care Unit, Royal Papworth Hospital NHS Foundation Trust, Cambridge, UK

[11]Addenbrooke's Hospital, Cambridge University Hospitals NHS Foundation Trust, Cambridge Biomedical Campus, Cambridge, UK

[12]Intensive Care Unit, Addenbrooke's Hospital, Cambridge University Hospitals NHS Foundation Trust, Cambridge Biomedical Campus, Cambridge, UK

[13]Department of Infectious Diseases, Addenbrooke's Hospital, Cambridge University NHS Hospitals Foundation Trust, Cambridge, UK

[14]Department of Microbiology, Addenbrooke's Hospital, Cambridge University NHS Hospitals Foundation Trust, Cambridge, UK

[15]Department of Renal Medicine, Addenbrooke's Hospital, Cambridge University Hospitals NHS Foundation Trust, Cambridge, UK

[16]Africa Health Research Institute, Durban, South Africa
[17]Clinical Genetics, Addenbrooke's Hospital, Cambridge University Hospitals NHS Foundation Trust, Cambridge, UK
[18]Department of Clinical Neurosciences, School of Clinical Medicine, University of Cambridge, Cambridge Biomedical Campus, Cambridge, UK
[19]MRC Toxicology Unit, Gleeson Building, Tennis Court Road, Cambridge, UK
[20]University of Cambridge, Cambridge, UK
[21]The Francis Crick Institute, London, UK
[22]Public Health England, Clinical Microbiology and Public Health Laboratory, Cambridge, UK
[23]Department of Clinical Neurosciences, School of Clinical Medicine, University of Cambridge, Cambridge Biomedical Campus, Cambridge, UK
[24]Medical Research Council Mitochondrial Biology Unit, Cambridge Biomedical Campus, Cambridge, UK
[25]Global and Tropical Health Division, Menzies School of Heath Research and Charles Darwin University, Darwin, Northern Territory, Australia
[26]Heart and Lung Research Institute, Cambridge Biomedical Campus, Cambridge, UK
[27]Department of Rheumatology, Addenbrooke's Hospital, Cambridge University Hospitals NHS Foundation Trust, Cambridge, UK
[28]Cambridge Cancer Trials Centre, Addenbrooke's Hospital, Cambridge University Hospitals NHS Foundation Trust, Cambridge, UK
[29]Department of Paediatrics, University of Cambridge, Cambridge Biomedical Campus, Cambridge, UK
[30]Patient Safety, Addenbrooke's Hospital, Cambridge University Hospitals NHS Foundation Trust, Cambridge, UK
[31]Clinical Research Network: Eastern, Addenbrooke's Hospital, Cambridge University Hospitals NHS Foundation Trust, Cambridge, UK
[32]Institute of Metabolic Science, Addenbrooke's Hospital, Cambridge University Hospitals NHS Foundation Trust, Cambridge, UK
[33]NHS Blood and Transplant, Cambridge, UK
[34]Cambridge Institute for Medical Research, Biomedical Campus, Hills Rd, Cambridge UK
